# Comparative efficacy of different ultrasound-guided ablation for the treatment of benign thyroid nodules: Systematic review and network meta-analysis of randomized controlled trials

**DOI:** 10.1371/journal.pone.0243864

**Published:** 2021-01-20

**Authors:** Linye He, Wanjun Zhao, Zijing Xia, Anping Su, Zhihui Li, Jingqiang Zhu

**Affiliations:** 1 Department of Thyroid & Parathyroid Surgery, West China Hospital, Sichuan University, Chengdu, Sichuan Province, China; 2 Department of Rheumatology and Immunology, West China Hospital, Sichuan University, Chengdu, China; MSc, PhD, Universidad de Antioquia, COLOMBIA

## Abstract

**Background:**

Percutaneous ablation is currently deemed an additionally treatment option for benign thyroid nodules in the world, but possibly different effect among the ablation modalities is not clear. So we aim to evaluate the efficacy and complications of thermal/chemical ablation by network meta-analysis.

**Materials and methods:**

In the network meta-analysis, PubMed, EMBASE and the Cochrane Library databases were searched from 1980 to 2020. Studies of adults with thyroid benign nodules under percutaneous ablation therapy were included. Percentage mean volume change, symptom score change, cosmetic score change and complications were evaluated by network meta-analysis.

**Results:**

In the network meta-analysis, Radiofrequency Ablation(RFA) with 2 treatment sessions group was associated with the highest reduction for the mean volume change during 6-month follow-up (MD = 79.09 and 95% CrI:48.23–89.94). There is no significant difference in the incidence of complications. Subgroup analysis showed that 2 sessions of Radiofrequency Ablation (RFA) ranks the highest probability (surface under the cumulative ranking curve (SUCRA) values 77.9) of being the most efficacious treatment for solid or predominantly solid benign nodules. Ethanol ablation (EA) ranked first (SUCRA value 81.1) in the treatment for cyst or predominantly cyst benign nodules.

**Conclusion:**

RFA appears to be superior to other US-guided percutaneous ablation in reducing benign thyroid nodule volume during short- and long-term follow-up. In the subgroup analysis, RFA with 2 treatment sessions showed the most significant effectiveness for solid benign thyroid nodules and EA showed more effectiveness to decrease the volume of cyst benign thyroid nodules.

## Introduction

Thyroid nodule (TN) is one of the most common endocrine lesions and has been increasingly detected in approximately 34–52% of the general population in the past two decades due to the widespread use of the high frequency ultrasound (US) [1]. The standard therapy for palpable TNs with subjective symptoms or cosmetic problems has been surgical excision rather than radioiodine or levothyroxine therapy [2], but major complications(such as hypothyroidism, transient or permanent hypoparathyroidism and recurrent laryngeal nerve injury) are observed in 2–7% of patients after treatment [3,4], which seriously affect the quality of patients’ life.

In recent years, US-guided minimally invasive techniques have been widely used for treatment of benign thyroid nodules [5–7]. According to the guideline of image-guided thyroid ablation in Europe and Asia, chemical and thermal ablation procedures have been proposed as the common modalities for non-surgical treatment for benign thyroid nodules [8–12]. Chemical ablation, including ethanol (EA) and polidocanol (PA) ablation, has been shown to be effective for primary and recurrent cystic benign thyroid nodules [13–15]. Moreover, EA was also proposed as an alternative therapy to surgery and radioiodine for large hyperfunctioning nodules in the clinical practice guideline of German and Korea [10,11]. Thermal ablation, including radiofrequency ablation (RFA) (which is generally used by monopolar electrodes during the procedure), laser ablation (LA), high-intensity focused ultrasound (HIFU) and microwave ablation (MWA), were widely evolved in the management of benign thyroid nodules complaining of symptomatic or cosmetic problems [16–18]. According to the current clinical practice guidelines for benign thyroid nodules, LA and RFA were recommended as the first-line thermal ablation treatment modalities while MWA was considered as a second-line procedure in patients who are not suitable for or decline other thermal ablation procedures [8–10]. However, some recommendation for the treatment of benign thyroid nodules were with low or very low quality of evidence and many studies had only made comparative analysis of two modalities [10–12]. Recently, a meta-analysis showed that RFA had better effect in reducing benign solid thyroid nodule volume than LA despite the smaller number of treatment sessions [19]. However, which kind of percutaneous ablation can superiorly have better effect on solid or cyst thyroid nodules in short- and long-term follow-up respectively is still controversially debated.

With the presented studies, we aimed to systematically review the literature of US-guided ablation for solid/cyst benign thyroid nodules and made a network meta-analysis to evaluate the efficacy and complications of different ablation therapies.

## Materials and methods

Our study was approved by the Ethics Committee of West China Hospital, Sichuan University and was conducted in compliance with the Health Insurance Portability and Accountability Act of 1996. The protocol of this meta-analysis was registered with the prospective register of systematic reviews, PROSPERO (identification code CRD42020150153). Supplemental material to this manuscript is publicly shared in an online repository [20].

### Study objectives

Our primary aim was to identify the percentage mean change in benign thyroid nodule volume during 6- and 12-month follow-up after US-guided percutaneous ablation (RFA, LA, HIFU and EA). The secondary aim was to identify symptom score and cosmetic score change during 6-month follow-up after operation. Overall complication was also evaluated during the study. Symptom score were defined as one that, after treatment, patients were asked to rate their symptoms on a 10-cm visual analog scale (scale, 0–10), and the physician recorded the cosmetic score as follows (1, no palpable mass; 2, no cosmetic problem but a palpable mass; 3, cosmetic problem on swallowing only; and 4, readily detected cosmetic problem at all times). Major and minor complications were as defined by the Society of Interventional Radiology [21] and a recent classification [22]. Major complication was regarded as an unexpected event which leads to substantial morbidity and disability, which also increases the level of care. All other unexpected adverse events should be regarded as minor. Subgroup outcomes were identified as the percentage mean change in solid/cyst benign thyroid nodule volume during 6- month follow-up after US-guided percutaneous ablation.

### Study selection

A decision regarding the inclusion of studies in the analyses was made by two independent reviewers (HLY and ZWJ) based on the predefined inclusion criteria. Studies were selected after a two-level screening. First, we screened the titles and abstracts of the identified studies. Second, we reviewed the full texts. Discrepancies between the reviewers were resolved through discussions.

Studies were included if they fulfilled the following inclusion criteria:(1) Randomized controlled trials; (2) complete follow-up data about the percentage mean change in benign thyroid nodule volume, symptom score, cosmetic score and complications during 6-month or more follow-up after US-guided percutaneous ablation (RFA, LA, HIFU, MWA and EA).

Inclusion criteria for patients were as following: (1) age older than 18; (2) presence of a solid or cyst thyroid nodule with cosmetic or compressive symptoms; (3) confirmation of benign findings in US-guided core needle or fine-needle aspiration (FNA) biopsies;(4) no history of radioiodine therapy or thermal/chemical ablation, no previous neck or trunk external beam radiotherapy, or refusal of or ineligibility for surgery. Types of interventions: Interventions comprised US-guided percutaneous ablations including RFA, LA, EA, MWA and HIFU. Control groups included patients with no treatment.

### Database and literature sources

A systematic review of randomized controlled trials reporting on the efficacy of ablation therapies for patients with benign thyroid nodules in English was conducted following Preferred Reporting Items for Systematic Reviews and Meta-Analyses [23] and Cochrane guidelines [24]. The time period was up to July 2020. Manual searching of reference lists from original articles and previous meta-analyses was also performed.

A search of Embase, Pubmed and the Cochrane library was conducted by 2 investigators using the following keywords: Thyroid benign nodule, Randomized controlled trial, Radiofrequency ablation, Ethanol ablation, Laser ablation, Microwave ablation and High-intensity focused ultrasound ablation (**S1 Table**). After the initial electronic search, we evaluated the identified studies and performed a manual search using Google Scholar. To identify unpublished or ongoing studies, we searched the World Health Organization International Clinical Trials Registry Platform and the ClinicalTrials.gov website. The articles identified were assessed individually for inclusion in the analysis.

### Data extraction and quality assessment

Two reviewers extracted the relevant information from the included trials using a predefined data extraction sheet. Any disagreement unresolved by the discussion was resolved in consultation with a third reviewer. The following variables were extracted from the studies: (1) Mean and standard deviation of the percentage mean change in benign thyroid nodule volume, symptom score change and cosmetic score as continuous variables, and dichotomous variables including the incidence of overall complication; (2) Demographic and clinical characteristics, such as age, sex, and number of patients in the different percutaneous ablation therapy; (3) First author, country, and year of publication; (4) Method of assessment. If the above variables were not found in the articles, we requested the data from their authors via email.

The two reviewers independently assessed the methodological quality of each study by using the Cochrane Collaboration’s tool for assessing the risk of bias (Review Manager Version 5.3, The Cochrane Collaboration, Oxford, UK). Any disagreements between the reviewers were resolved through discussions or by the third reviewer. Included RCTs were classified into 1 of 3 categories: low risk, high risk, or unclear risk. The extracted data were verified independently.

### Statistical analysis

The primary outcome was the percentage mean change in benign thyroid nodule volume and the outcome measure was its mean difference(MD) with 95% confidence interval (CI). For direct comparisons, standard pairwise meta-analysis was performed using the inverse variance DerSimonian-Laird random effects model [25]. If a direct comparison was based on 2 or more studies, between-study heterogeneity, which represents the extent of variation among the intervention effects observed in different studies, was quantified using the I-squared statistic. Heterogeneity was considered low, moderate, or high for I-squared values <25%, 25% to 50%, and >50%, respectively [26].

For indirect and mixed comparisons, random effects network meta-analysis using Markov chain Monte Carlo simulations was carried out within a frequentist setting [26,27]. In the Bayesian network meta-analysis, we used non-informative (vague), prior distributions that allow data to drive the posterior distributions. The achievement of convergence was evaluated using the Brooks-Gelman-Rubin statistics. The results of network meta-analyses(NMA) with effect sizes (MD) and 95% CrI were summarized.

The plausibility of the transitivity assumption was assessed based on the design characteristics and the methodology of the studies included in the NMA, as recommended [28]. Inconsistency has been investigated using a design-by-treatment interaction model, which addresses both loop and design inconsistencies [29], In each loop, we evaluated the inconsistency factor (IF) as the absolute difference (95% confidence interval [CI]) and using z-test between the direct and indirect estimates for each paired comparison in the loop. The IF is the logarithm of the ratio of two odds ratios (RoRs) from direct and indirect evidence in the loop; RoRs close to 1 indicate that the two sources are in agreement. Additionally, subgroup analyses were performed to evaluate the robustness of the findings. Network meta-analysis also provides a ranking probability curve of each treatment (rankogram) by calculating the probability of each arm achieving the best rank amongst all treatments. The surface under the cumulative ranking (SUCRA) line for each treatment, which equals 100 when a treatment is certain to be the best and 0 when a treatment is certain to be the worst, was used for treatment ranking. A higher SUCRA value was regarded as a better result for individual interventions [26,30].

All statistical tests were 2-sided. Statistical analysis and graph generation were performed with Stata 14.0 (Stata Corp, College Station, TX) and R statistical software, version 3.6 (R Foundation for Statistical Computing; https://www.R-project.org/). All articles were assessed for risk of bias by 2 investigators using the Cochrane Risk of bias tool [24] (RevMan, version 5.1, The Nordic Cochrane Centre: The Cochrane Collaboration, Copenhagen, Norway).

## Results

### Study characteristics

**Fig 1:**
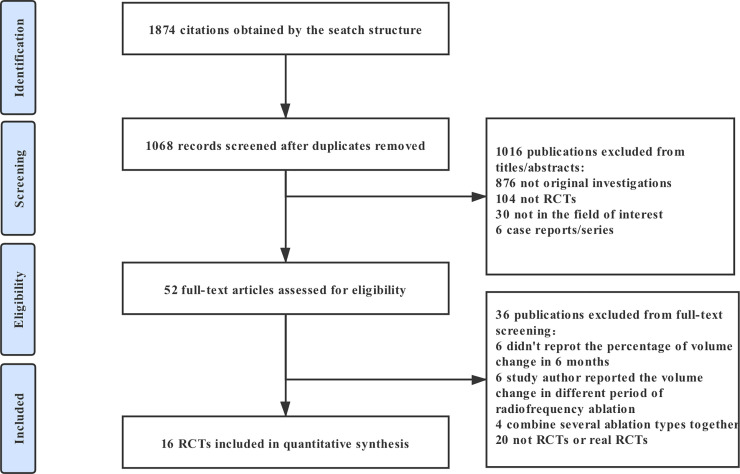
Flow chart of studies considered for inclusion.

52 full-text articles assessed for eligibility. Among the studies, 20 studies were retrospective studies, 6 reported mean volume change in less than 6 months, 6 studies reported the percentage of volume change in different period of RFA while the nature of the nodules was not identified specifically and 4 studies combined several ablation types together. A total of 16 RCTs including 843 patients were eligible (Fig 1) [31–46]. Studies have compared the following treatments: RFA, LA, HIFU and EA. The main features of these studies are reported in **Table 1**. In terms of the demographic characteristics of the patients in the included studies, all of the eligible studies were two-armed trials (RFA vs control group, LA vs control group, EA vs control group, HIFU vs control group, RFA vs EA, RFA vs RFA with 2 treatment sessions, LA vs LA with 3 treatment sessions and EA vs EA with 3 treatment sessions). In the RCTs, there were 3 studies reported to treat with cyst or predominantly cyst benign thyroid nodules [33,35,37], 13 studies to treat with solid or predominantly solid benign nodules [31,32,34,36,38–46].

**Table 1:** Nodule characteristics in the included studies.

First author	Year	Country	No. of patients	Interventions	Mean Age(Y)	Gender (Male/Female)	Follow-up (Month)	Symptom score (baseline)	Initial Volume(mL)	Treatment session	Study
Maurilio Deandrea	2015	Italy	40	RFA	46.9 ± 13.68	6/34	6	3.6 ± 1.9	15.1 ± 3.1	1	Multi-center
			40	Control	57.4 ± 12.55	2/38	6	3.5 ± 1.61	14.4 ± 3.3	1	
Jung Hwan Baek	2015	Korea	22	RFA	49.8 ± 13.5	3/19	6	2.9 ± 1.8	8.6 ± 9.4	1	Single Center
			24	EA	50.8 ± 15.2	6/18	6	4.0 ± 1.7	14.7 ± 13.7	1	
Roberto Cesareo	2015	Italy	42	RFA	56 ± 14	15/27	6	2.8 ± 3.3	24.5 ± 19.6	1	Single Center
			42	Control	53 ± 12	18/24	6	2.7 ± 3	27.5 ± 22.1	1	
Enrico Papini	2014	Italy	101	LA	51.5 ± 13.7	16/85	36	N/A	N/A	1	Single Center
			99	Control	54.7 ± 13.7	23/76	36	N/A	N/A	1	
Laurence Leenhardt	2013	France	21	HIFU	N/A	N/A	6	N/A	N/A	1	Single Center
			11	Control	N/A	N/A	6	N/A	N/A	1	
Jin Yong Sung	2013	Korea	25	EA	45.0 ± 10.9	2/23	6	3.4 ± 2.0	12.2 ± 11.0	1	Single Center
			25	RFA	44.9 ± 10.6	3/22	6	3.5 ± 2.2	9.3 ± 11.7	1	
Helle Døssing	2013	Denmark	22	LA	49(39,56)	5/17	6	N/A	11.8	1	Single Center
			22	Control	49(40,56)	9/13	6	N/A	10	1	
Jung Yin Huh	2012	Korea	15	RFA	37.5 ± 11.5	2/13	6	5.4 ± 1.7	N/A	1	Single Center
			15	RFA2	37.7 ± 9.8	0/15	6	5.3 ± 1.8	N/A	2	
Faggiano	2012	Italy	20	RFA	58.3 ± 4.3	4/16	12	5.4 ± 0.3	N/A	1	Single Center
			20	Control	62.1 ± 3.1	5/15	12	5.3 ± 0.3	N/A	1	
Jung Hwan Baek	2010	Korea	15	RFA	40.87 ± 11.08	3/12	6	3.13 ± 1.51	7.5 ± 4.9	1	Single Center
			15	Control	47.47 ± 9.01	3/12	6	3.33 ± 0.90	6.9 ± 4.0	1	
Enrico Papini	2007	Italy	21	LA	44.9 ± 5.1	3/18	12	N/A	11.7 ± 5.1	1	Single Center
			20	Control	47.1 ± 7.7	2/18	12	N/A	12.1 ± 3.9	1	
Gambelunghe	2006	Italy	13	LA	63	11/2	7	N/A	8.2	1	Single Center
			13	Control	70	10/3	7	N/A	8.1	1	
Helle Døssing	2006	Denmark	15	LA	46 ± 7	1/14	6	4.5 ± 2.0	10.1 ± 4.3	1	Single Center
			15	LA3	45 ± 12	0/15	6	4.5 ± 2.3	10.7 ± 9.0	3	
Helle Døssing	2005	Denmark	15	LA	47(43,52)	0/15	6	3.0 ± 2.2	8.2(6.1,11.9)	1	Single Center
			15	Control	46(41,51)	0/15	6	4.0 ± 2.1	7.5(5.1,13.8)	1	
BENNEDBÆK, F. N	1999	Denmark	30	EA	42.6 ± 10.6	1/29	6	4.6 ± 0.40	9.9 ± 5.7	1	Single Center
			30	EA3	42.7 ± 10.0	0/30	6	4.0 ± 0.43	9.4 ± 4.2	3	
BENNEDBÆK, F. N	1998	Denmark	25	EA	46(41,52)	3/22	12	N/A	9.2(7.2,11.6)	1	Single Center
			25	Control	41(37,45)	1/24	12	N/A	7.1(4.9,10.8)	1	

**RFA**: Radiofrequency Ablation with single treatment session; **RFA2**: Radiofrequency Ablation with 2 treatment session; **EA**: Ethanol Ablation with single treatment session; **EA3**: Ethanol Ablation with 3 treatment session; **LA**: Laser ablation with single treatment session; **LA3**: Laser ablation with 3 treatment session**. RCT**: Randomized Controlled Trial.

### Quality assessment of trials and evidence grading

For randomization, all of the 16 studies in our study was randomized. The risk of bias was high for blinding of participants and personnel in 16 trials because of the limitation of the intervention in the trials; Concealment of treatment allocation in 14 trials. Blinding of outcome assessment in 14 trials. None of the studies selectively reported of outcomes in three trials (**S1 Fig**).

Before conducting the network meta-analyses, we evaluated the transitivity assumption by examining the comparability of the risk of bias as a potential treatment-effect modifier across comparisons. After confirming that the transitivity assumption was not violated, we conducted the network meta-analyses and consistency assessments. In the network meta-analyses, there was no evidence of violation of the transitivity assumption, based on the observations that the control group (only followed-up) was reasonably consistent across trials and participants could in principle be randomized to any of the treatments being compared in the network. Moreover, for the primary outcomes, the design-by-volume change interaction model showed no evidence of statistically significant inconsistency (P = 0.25) and there was also no significant inconsistency in the analysis for overall complications(P = 0.74). The quality of evidence assessed by GRADE analysis varied from moderate to very high for the meta-analyses estimates (**S2 Fig**). Funnel plot analyses did not indicate any evident risk of publication bias in terms of outcomes (percentage mean change in benign thyroid nodule volume, symptom score change, cosmetic score change and overall complication) and subgroup analysis (**S3 Fig**). Finally, there was no significant differences between direct and indirect estimates in closed loops that allowed assessment of network coherence (**S4A and S4B Fig**).

### Pairwise meta-analysis

Results of standard pairwise meta-analysis of direct comparisons in primary outcomes and subgroup analysis were fully reported in Table 2. Direct meta-analysis of primary outcomes(percentage mean volume change during 6-month follow-up) was feasible for the following comparisons: RFA versus control group (4 trials, N = 234); LA versus control group (5 trials, N = 314); EA versus control group (1 trials, N = 50); HIFU versus control group (1 trials, N = 32); RFA versus EA(2 trials, N = 96); RFA with single treatment session versus RFA with 2 treatment session (1 trials, N = 30); LA with single treatment session versus LA with 3 treatment session (1 trials, N = 30); EA with single treatment session versus EA with 3 treatment session (1 trials, N = 60). RFA, LA, EA and HIFU were associated with a statistically significant outcomes compared with control group (summary MD = 73.48, 95%CI:68.40–78.56 for RFA; MD = 35.49, 95%CI: 16.25–54.72 for LA; MD = 38, 95%CI:36.66–39.34 for EA; MD = 44.90, 95%CI: 16.56–73.24 for HIFU).

**Table 2:** Results of pooled outcomes in the network meta-analysis and pairwise meta-analysis.

Comparison	Pairwise meta-analysis Mean Difference (95% CI)	Network meta-analysis Mean Difference (95% CrI)	No. of participants	No. of trials	P-value	Heterogeneity I^2^
***Percentage mean change in benign thyroid nodule volume during 6-month follow-up***						
RFA vs Control group	73.48(68.40, 78.56)	79.09(48.23, 89.94)	234	4	<0.00001	66%
LA vs Control group	35.49(16.25, 54.72)	60.83(42.10, 79.55)	314	5	0.0003	100%
HIFU vs Control group	44.90(16.56, 73.24)	44.90(3.82, 93.62)	32	1	0.002	0%
EA vs Control group	38.00(36.66, 39.34)	37.19(-3.78, 78.17)	50	1	<0.00001	0%
RFA2 vs RFA	8.10(0.34, 15.86)	7.95(-33.90, 49.80)	30	1	0.04	0%
RFA vs EA	-3.22(-5.82, -0.62)	4.68(-37.87, 47.23)	96	2	0.02	45%
LA3 vs LA	13.00(2.17, 23.83)	12.93(-29.61, 55.47)	30	1	0.02	0%
EA3 vs EA	4.70(-6.15, 15.55)	4.68(-37.87, 47.23)	60	1	0.4	0%
***Percentage mean change in benign thyroid nodule volume during 12-month follow-up***						
RFA vs Control group	74.60(71.12, 78.08)	88.40(47.35, 129.45)	40	1	<0.00001	0%
LA vs Control group	19.02(-7.52, 45.56)	79.20(38.04, 120.36)	241	2	0.16	97%
EA vs Control group	38.00(36.66, 39.34)	54.29(23.87, 87.71)	50	1	<0.00001	0%
***Percentage mean change in solid or predominantly solid benign thyroid nodule volume during 6-month follow-up***						
RFA vs Control group	73.48(68.40, 78.56)	69.06(47.64, 90.49)	234	4	<0.00001	66%
LA vs Control group	42.41(34.90, 49.92)	59.73(38.20, 81.26)	270	4	<0.00001	69%
HIFU vs Control group	44.90(16.56, 73.24)	44.90(-4.80, 94.60)	32	1	0.002	0%
EA vs Control group	38.00(36.66, 39.34)	37.96(-4.57, 80.49)	50	1	<0.00001	0%
RFA2 vs RFA	8.10(0.34, 15.86)	7.94(-35.05, 50.92)	30	1	0.04	0%
LA3 vs LA	13.00(2.17, 23.83)	12.93(-30.73, 56.58)	30	1	0.02	0%
EA3 vs EA	4.70(-6.15, 15.55)	4.68(-38.99, 48.35)	60	1	0.4	0%
***Percentage mean change in cyst or predominantly cyst benign thyroid nodule volume during 6-month follow-up***						
RFA vs EA	3.22(0.62, 5.82)	-1.49(-10.96, 7.99)	96	2	0.02	45%
LA vs Control group	45.00(35.80, 54.20)	45.00(35.80, 54.20)	44	1	<0.00001	0%

**95% CI**: 95% Confidence Intervals; **95% CrI**: 95% Credible Intervals; **RFA**: Radiofrequency Ablation with single treatment session; **RFA2**: Radiofrequency Ablation with 2 treatment session; **EA**: Ethanol Ablation with single treatment session; **EA3**: Ethanol Ablation with 3 treatment session; **LA**: Laser ablation with single treatment session; **LA3**: Laser ablation with 3 treatment session.

There were also statistically significant difference between RFA and RFA with 2 treatment sessions, LA and LA with 3 treatment sessions, RFA and EA (MD = 8.10, 95%CI: 0.34–15.86, MD = 13.00, 95%CI: 2.17–23.83, MD = -3.22, 95%CI: -5.82- -0.52, respectively) while EA vs EA with 3 treatment sessions resulted in a nonsignificant effect on volume change (**Table 2**). For long-term mean volume reduction in thyroid benign nodules (12-month follow-up), RFA and EA were significantly better than the control group (MD = 74.60, 95%CI: 71.12–78.08 and MD = 38.00, 95%CI: 36.66–39.34) (**Table 2**).

### Network meta-analysis

**Fig 2:**
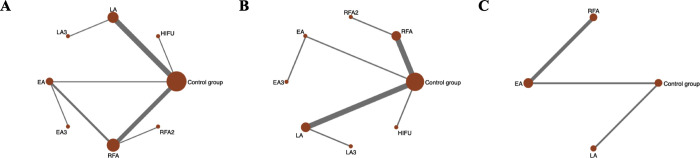
Evidence structure of eligible comparisons. (A)Network diagrams of comparisons for the percentage mean change in benign thyroid nodule volume during 6-month follow-up after thermal/chemical ablation. (B) Network diagrams of comparisons for the percentage mean change in solid or predominantly solid benign thyroid nodule volume after thermal/chemical ablation. (C) Network diagrams of comparisons for the percentage mean change in cyst or predominantly cyst benign thyroid nodule volume after thermal/chemical ablation. **RFA**: Radiofrequency Ablation with single treatment session**; RFA2**: Radiofrequency Ablation with 2 treatment session**; EA:** ethanol ablation with single treatment session**; EA3**: ethanol ablation with 3 treatment session; **LA**: Laser Ablation with single treatment session**; LA3**: Laser Ablation with 3 treatment session. **HIFU**: High-Intensity Focused Ultrasound.

A network meta-analysis was conducted to investigate the following treatments in randomized controlled trials: RFA, RFA2, LA, LA3, EA, EA3, HIFU and control group for the primary outcome, **(Fig 2A**). LA and the control are the two most frequent comparators across the studies. 8 of 28 pairwise comparisons had direct evidence. **S6 Fig** summarizes the contribution of direct comparisons in determining the network meta-analysis estimates for mixed and indirect evidence, which demonstrates that schedules including RFA, LA and EA comparing with control group had the highest weight in the network meta-analysis in different results.

In the results of network meta-analysis for primary outcome(**Table 3**), RFA with single and 2 treatment sessions (MD = 62.52, 95% CrI:23.05–81.06; MD = 79.09 and 95% CrI:48.23–89.94), LA with single and 3 treatment sessions (MD = 60.83, 95% CrI:42.10–79.55 and MD = 73.76, 95% CrI:27.29–120.23) and HIFU (MD = 33.90, 95% CrI:3.82–79.55) significantly reduced the nodule volume during 6-month follow-up compared to the control group while EA with single and 3 treatment sessions didn’t make a significant result when compared to control group (MD = 37.19, 95% CrI: -3.78–78.17 and MD = 41.87, 95% CrI: -17.18–100.93). Ranking analysis performed with surface under the cumulative ranking curve(SUCRA) showed RFA with 2 treatment sessions and LA with 3 treatment sessions (SUCRA score are 78.6 and 73.8) rank first and second, respectively. Subsequently, the other treatments were ranked as follows: RFA with single treatment sessions (72.0), LA with single treatment sessions (58.4), HIFU (41.7), EA with 3 treatment sessions (40.8), EA with single treatment sessions (32.4), and control group (0.5) (**Table 5**). For long-term mean volume reduction in thyroid benign nodules, network meta-analysis suggested that compared with the control group, RFA were associated with significantly better effect. (MD = 88.40, 95%CI: 47.35–129.45; SUCRA = 84.3), followed by LA (MD = 79.20, 95%CI: 38.04–120.36; SUCRA = 73.6) and EA (MD = 54.29, 95%CI: 23.87–84.71, SUCRA = 42.1) (**Tables 3 and 5**). **The cumulative ranking curve of the outcomes were showed in S5 Fig.**

**Table 3:** Results of network meta-analysis for percentage mean volume change of benign thyroid nodules after percutaneous ablation therapies in 6- and 12-month follow-up and symptom score and cosmetic score change of benign thyroid nodules after percutaneous ablation therapies in 6 months.

	Outcome	RFA2	LA3	RFA	LA	HIFU	EA3	EA	Control group
		MD (95%Crl)	MD (95%Crl)	MD (95%Crl)	MD (95%Crl)	MD (95%Crl)	MD (95%Crl)	MD (95%Crl)	MD (95%Crl)
**RFA2**	**Percentage Mean Volume Change (6-month)**								
	**Percentage Mean Volume Change (12-month)**								
	**Symptom Score Change**								
	**Cosmetic Score Change**								
**LA3**	**Percentage Mean Volume Change (6-month)**	3.28 (-62.63,69.19)							
	**Percentage Mean Volume Change (12-month)**								
	**Symptom Score Change**	0.91 (-4.39,6.21)							
	**Cosmetic Score Change**	***2*.*21 (0*.*50*,*12*.*93)***							
**RFA**	**Percentage Mean Volume Change (6-month)**	7.95 (-33.90,49.80)	4.67 (-46.27,55.60)						
	**Percentage Mean Volume Change (12-month)**								
	**Symptom Score Change**	-0.10 (-1.82,1.62)	-1.01 (-6.02,4.00)						
	**Cosmetic Score Change**	***1*.*80 (0*.*04*,*3*.*56)***	-0.41 (-10.99,10.99)						
**LA**	**Percentage Mean Volume Change (6-month)**	16.21 (-34.15,66.57)	12.93 (-29.61,55.47)	8.26 (-19.77,36.30)					
	**Percentage Mean Volume Change (12-month)**			9.20 (-48.93,67.33)					
	**Symptom Score Change**	0.41 (-4.04,4.85)	-0.50 (-3.39,2.39)	0.51 (-3.59,4.61)					
	**Cosmetic Score Change**	***2*.*41 (0*.*67*,*12*.*49)***	0.20 (-3.44,3.84)	0.61 (-9.31,10.54					
**HIFU**	**Percentage Mean Volume Change (6-month)**	32.14 (-35.38,99.65)	28.86 (-38.47,96.18)	24.19 (-28.81,77.18)	15.93 (-36.27,68.12)				
	**Percentage Mean Volume Change (12-month)**								
	**Symptom Score Change**								
	**Cosmetic Score Change**								
**EA3**	**Percentage Mean Volume Change (6-month)**	35.16 (-40.15,110.47)	31.88 (-43.26,107.03)	27.22 (-35.41,89.84)	18.95 (-43.00,80.91)	3.03 (-73.53,79.59)			
	**Percentage Mean Volume Change (12-month)**								
	**Symptom Score Change**	-0.40 (-2.48,1.67)	-1.31 (-6.46,3.83)	-0.30 (-1.46,0.85)	-0.81 (-5.07,3.45)				
	**Cosmetic Score Change**	***2*.*78 (0*.*56*,*4*.*99)***	0.56 (-10.07,11.20)	0.98 (-0.37,2.32)	0.36 (-9.63,10.36)				
**EA**	**Percentage Mean Volume Change (6-month)**	39.84 (-22.31,101.99)	36.56 (-25.39,98.52)	31.90 (-14.07,77.86)	23.63 (-21.42,68.69)	7.71 (-55.95,71.37)	4.68 (-37.87,47.23)		
	**Percentage Mean Volume Change (12-month)**			34.11 (-16.98,85.20)	24.91 (-26.27,76.09)				
	**Symptom Score Change**	-0.40 (-2.47,1.66)	-1.31 (-6.46,3.83)	-0.30 (-1.46,0.85)	-0.81 (-5.07,3.44		0.00 (-0.09,0.09)		
	**Cosmetic Score Change**	***2*.*32 (0*.*38*,*4*.*26)***	0.11 (-10.49,10.70)	0.52 (-0.30,1.34)	-0.09 (-10.04,9.85)		-0.45 (-1.67,0.76)		
**Control Group**	**Percentage Mean Volume Change (6-month)**	***69*.*09 (48*.*23*,*89*.*94)***	***73*.*76 (27*.*29*,*120*.*23)***	***62*.*52 (23*.*05*,*81*.*06)***	***60*.*83 (42*.*10*,*79*.*55)***	44.90 (-3.82,93.62)	41.87 (-17.18,100.93)	37.19 (-3.78,78.17)	
	**Percentage Mean Volume Change (12-month)**			***88*.*40 (47*.*35*,*129*.*45)***	***79*.*20 (38*.*04*,*120*.*36)***			***54*.*29 (23*.*87*,*84*.*71)***	
	**Symptom Score Change**	***2*.*51 (0*.*75*,*4*.*27)***	1.60 (-3.40,6.60)	***2*.*61 (2*.*22*,*2*.*99)***	2.10 (-1.98,6.18)		***2*.*91 (1*.*69*,*4*.*13)***	***2*.*91 (1*.*70*,*4*.*13)***	
	**Cosmetic Score Change**	***3*.*30 (1*.*41*,*5*.*20)***	***1*.*09 (0*.*46*,*11*.*64)***	***1*.*50 (0*.*80*,*2*.*20)***	0.89 (-9.01,10.79)		0.53 (-0.84,1.89)	***0*.*98 (0*.*01*,*1*.*95)***	

**NOTE.** Indirect comparison values are below the diagonal. For values below the diagonal, values greater than 0 reflect increased efficacy by the treatment specified in the top row. Bold numbers denote a statistically significant difference in efficacy of one treatment. **RFA**: Radiofrequency Ablation with single treatment session**; RFA2**: Radiofrequency Ablation with 2 treatment session**; EA:** ethanol ablation with single treatment session**; EA3**: ethanol ablation with 3 treatment session; **LA**: Laser Ablation with single treatment session**; LA3**: Laser Ablation with 3 treatment session. **HIFU**: High-Intensity Focused Ultrasound.

In terms of secondary outcome, an analysis was conducted grouping together all the eligible studies. Network meta-analysis showed significant symptom score advantage of EA with single or 3 treatment sessions and RFA with single or 2 treatment sessions compared with control group (MD = 2.91, 95% CrI: 1.70–4.13; MD = 2.91, 95% CrI: 1.69–4.13; MD = 2.61, 95% CrI:2.22–2.99; MD = 2.51, 95% CrI: 0.75–4.27, respectively) (**Table 3**). Ranking analysis revealed that EA with single treatment session was superior treatment followed by EA with 3 treatment sessions then RFA with single treatment sessions, RFA with 3 treatment sessions, LA with single or 3 treatment sessions and control group (SUCRA values 69.3, 69.2, 56.3, 55.9, 51.1, 41.3 and 7.0, respectively) (**Table 5**). In the analysis of cosmetic score change during 6-month follow-up, RFA with single or 2 treatment sessions, LA with 3 treatment sessions and EA with 3 treatment session (MD = 1.50, 95% CrI: 0.80–2.20; MD = 3.30, 95% CrI: 1.41–5.20; MD = 1.09, 95% CrI: 0.46–11.64; MD = 0.98, 95% CrI: 0.01–1.95, respectively) have significantly advantage compared to control group (**Table 3**). RFA with 2 treatment sessions also has significantly better effect on the cosmetic problems than the other 5 treatments (MD = 1.80, 95% CrI: 0.04–3.56; MD = 2.21, 95% CrI: 0.50–12.93; MD = 2.32, 95% CrI: 0.38–4.26; MD = 2.41, 95% CrI: 0.67–12.49; MD = 2.78, 95% CrI: 0.56–4.99, respectively). Ranking analysis revealed that RFA with 2 treatment sessions was superior treatment followed by RFA with single treatment session then LA with 3 treatment sessions, EA with single treatment session, LA with single treatment session, EA with 3 treatment sessions and control group (SUCRA values 88.2, 65.4, 41.0, 49.8, 47.8, 46.9, 33.7 and 18.2, respectively) (**Table 5**) As for complications of each therapies, the overall complications of the included studies were summarized in **S2 Table**. A total of 53 complications were reported. There was no significant publication bias noted for overall complications while heterogeneity was noted (**S3G Fig**). In the network analysis, All US-guided ablation therapy has no significantly incidence of overall complications compared to control group (**S3 Table**). Ranking analysis revealed that HIFU caused lower incidence of complications followed by RFA with single or 2 treatment sessions then EA and LA with single sessions and EA and LA with 3 treatment sessions (SUCRA values 79.5, 59.7, 55.8, 48.7, 35.1,23.2 and 16.5, respectively) (**Table 5**). All t**he cumulative ranking curve of the outcomes were showed in S5 Fig**.

### Subgroup analysis in pairwise and network meta-analysis

To analysis the subgroup of solid or cyst nodules, we repeated the network meta-analysis using primary outcomes as endpoints. Subgroup analysis for solid or predominantly solid benign nodules was conducted in 13 trials. Trials compared the following treatments: RFA with single or two treatment sessions, LA with single or three treatment sessions, EA with single or three treatment sessions and HIFU (**Fig 2B**). Patients had a significant volume change when treated with RFA with single or two treatment sessions (MD = 77.00, 95%CrI:28.99–125.01 and MD = 69.06, 95%CrI:47.64–90.49) and LA with single or three treatment sessions (MD = 72.66, 95%CrI:23.99–121.32 and MD = 59.73, 95%CrI:38.20–81.26) compared to control group (**Table 4**). In ranking analysis, 2 sessions of RFA showed the highest probability (SUCRA values 77.9) of being the most efficacious treatment, followed by three sessions of LA (74.0), single sessions of RFA (71.6), one session of LA (57.6), HIFU (41.6), three sessions of EA (41.3), one session of EA (33.6), and the control group (2.5) (**Table 5 and S5 Fig**).

**Table 4:** Results of network meta-analysis for percent mean volume change in the subgroup of solid or cyst benign thyroid nodules after percutaneous ablation therapies.

	Outcome	RFA2	LA3	RFA	LA	HIFU	EA3	EA	Control group
		MD (95%Crl)	MD (95%Crl)	MD (95%Crl)	MD (95%Crl)	MD (95%Crl)	MD (95%Crl)	MD (95%Crl)	MD (95%Crl)
**RFA2**	**Percentage Mean Volume Change (solid nodules)**								
	**Percentage Mean Volume Change (cyst nodules)**								
**LA3**	**Percentage Mean Volume Change (solid nodules)**	4.34 (-64.02,72.71)							
	**Percentage Mean Volume Change (cyst nodules)**								
**RFA**	**Percentage Mean Volume Change (solid nodules)**	7.94 (-35.05,50.92)	3.59 (-49.58,56.77)						
	**Percentage Mean Volume Change (cyst nodules)**								
**LA**	**Percentage Mean Volume Change (solid nodules)**	17.27 (-35.35,69.89)	12.93 (-30.73,56.58)	9.33 (-21.04,39.71)					
	**Percentage Mean Volume Change (cyst nodules)**			8.27 (-14.91,31.46)					
**HIFU**	**Percentage Mean Volume Change (solid nodules)**	32.10 (-37.00,101.20)	27.76 (-41.80,97.32)	24.16 (-29.96,78.28)	14.83 (-39.33,68.99)				
	**Percentage Mean Volume Change (cyst nodules)**								
**EA3**	**Percentage Mean Volume Change (solid nodules)**	34.36 (-43.22,111.95)	30.02 (-47.97,108.01)	26.43 (-38.17,91.02)	17.09 (-47.54,81.73)	2.26 (-76.37,80.90)			
	**Percentage Mean Volume Change (cyst nodules)**								
**EA**	**Percentage Mean Volume Change (solid nodules)**	39.04 (-25.10,103.18)	34.70 (-29.94,99.33)	31.10 (-16.52,78.73)	21.77 (-25.90,69.44)	6.94 (-58.47,72.36)	4.68 (-38.99,48.35)		
	**Percentage Mean Volume Change (cyst nodules)**			-1.49 (-10.96,7.99)	-9.76 (-31.00,11.48)				
**Control group**	**Percentage Mean Volume Change (solid nodules)**	***77*.*00 (28*.*99*,*125*.*01)***	***72*.*66 (23*.*99*,*121*.*32)***	***69*.*06 (47*.*64*,*90*.*49)***	***59*.*73 (38*.*20*,*81*.*26)***	44.90 (-4.80,94.60)	42.64 (-18.31,103.58)	37.96 (-4.57,80.49)	
	**Percentage Mean Volume Change (cyst nodules)**			***53*.*27 (31*.*99*,*74*.*55)***	***45*.*00 (35*.*80*,*54*.*20)***			***54*.*76 (35*.*61*,*73*.*90)***	

**NOTE.** Indirect comparison values are below the diagonal. For values below the diagonal, values greater than 0 reflect increased efficacy by the treatment specified in the top row. Bold numbers denote a statistically significant difference in efficacy of one treatment. **RFA**: Radiofrequency Ablation with single treatment session**; RFA2**: Radiofrequency Ablation with 2 treatment session**; EA:** ethanol ablation with single treatment session**; EA3**: ethanol ablation with 3 treatment session; **LA**: Laser Ablation with single treatment session**; LA3**: Laser Ablation with 3 treatment session. **HIFU**: High-Intensity Focused Ultrasound.

**Table 5:** SUCRA values and ranks of percentage mean volume changes, symptom score and cosmetic score.

Code	Treatments	Percentage Mean Volume Change (6-month)		Percentage Mean Volume Change (12-month)		Percentage Mean Volume Change (solid nodules)		Percentage Mean Volume Change (cyst nodules)		Symptom score Change		Cosmetic score Change		Overall Complication	
		SUCRA	Rank	SUCRA	Rank	SUCRA	Rank	SUCRA	Rank	SUCRA	Rank	SUCRA	Rank	SUCRA	Rank
1	Radiofrequency Ablation	**72.0**	**3**	**84.3**	**1**	**71.6**	**3**	**70.8**	**2**	**56.3**	**4**	**65.4**	**2**	**59.7**	**3**
2	Radiofrequency Ablation with 2 treatment sessions	**78.6**	**1**			**77.9**	**1**			**55.9**	**3**	**88.2**	**1**	55.8	4
3	Ethanol Ablation	32.4	7	**42.1**	**3**	33.6	7	**81.1**	**1**	45.2	6	**47.8**	**4**	48.7	5
4	Ethanol Ablation with 3 treatment sessions	40.8	6			41.3	6			69.2	2	33.7	6	23.2	7
5	Laser Ablation	58.4	4	**73.6**	**2**	**57.6**	**4**	**48.1**	**3**	**69.3**	**1**	46.9	5	35.1	6
6	Laser Ablation with 3 treatment sessions	**73.8**	**2**			**74.0**	**2**			51.1	5	**49.8**	**3**	16.5	8
7	High-Intensity Focused Ultrasound	41.7	5			41.6	5							**79.5**	**2**
8	Control	0.5	8	0	4	2.5	8	0	4	7.0	7	18.2	7	**81.5**	**1**

**Note:** Rank: Intervention with higher SUCRA values would have higher probability of being better treatment among the comparisons. SUCRA: surface under the cumulative ranking curve.

Subgroup analysis was conducted for 3 trials investigating cystic or predominantly cystic benign nodules with ablation therapies, whereas EA, LA and RFA all showed a borderline than control group. (MD = 54.76, 95%CrI: 35.61–73.90; MD = 53.27, 95% CrI:31.99–74.55; MD = 45, 95%CrI: 35.80–54.20) (**Table 4**). EA ranked first (SUCRA value 81.1) followed by RFA (70.8), LA (48.1) and control group (0) (**Table 5 and S5 Fig**).

## Discussion

In the previous American Thyroid Association (ATA) guidelines, percutaneous ablations were not recommended for the treatment of benign thyroid nodules except for surgery [47]. However, ablation therapies were reported to be an effective therapy for the benign thyroid nodules in many researches. Compared to the surgery, ablation techniques showed less minimally invasiveness and lower incidence of complications. According to the current recommendation of European Thyroid Association [8], adult patients with benign thyroid nodules that cause pressure symptoms and/or cosmetic concerns and decline surgery, image-guided thermal ablation should be considered as a cost- and risk-effective alternative option to surgical treatment or observation alone. Particularly, this meta-analysis demonstrated that most of percutaneous ablations including RFA, LA, and HIFU showed significant reduction in solid/cyst nodule volume at short- or long-term follow-ups; In the network meta-analysis, RFA revealed superior efficacy to the other US-guided ablations for volume reduction of benign thyroid nodules and lower incidence of overall complications while LA are devoid of improvement of compression symptom and cosmetic concerns. In the solid subgroup analysis, RFA with 2 treatment sessions showed the most significant effectiveness and EA showed the most effectiveness to decrease the mean volume of benign thyroid nodules for cyst nodules during 6-month follow-up.

At present, the prevailing mainstream view [48–50] was that laser or radiofrequency ablation were recommended for the treatment of solid or complex thyroid nodules that progressively enlarge with symptomatic or cause cosmetic concern, which was exactly in line with our study. Moreover, according to our network meta-analysis, RFA might have superior effect on solid or predominantly solid benign thyroid nodules than LA while less complication might be observed during the treatment of LA. However, there was still few solid evidence (including RCTs) showing which kind of percutaneous ablations took superior advantage and less complication when treated with benign thyroid nodules.

Previous studies of non-surgical treatment for benign thyroid nodules have shown that several factors were related to nodule-volume reduction, such as nodular nature, treatment session and ablation technique. In Eun Ju Ha’s network meta-analysis [19], RFA appeared to be superior to LA in reducing benign solid thyroid nodule volume, with which our study has found the same conclusion while we have included more studies. Besides, subgroup analysis was performed to assess ablation efficacy for solid and cystic nodules. In the subgroup analysis, thermal ablation has more superior efficient on volume change than chemical therapy for solid or predominantly solid benign nodules. In the meanwhile, chemical ablation presented a significant efficacy in the ablation of cystic nodules. Long-term follow-up studies by Cho et al [51] have visualized the importance of the length of the follow-up period, they reported that long-term follow-up analysis of more than 3 years showed RFA was superior to LA for treating benign thyroid nodules, with less regrowth and less delayed surgery. However, this study included both retrospective studies and randomized controlled trials, which may cause publication bias during the meta-analyses. Therefore, we strictly matched for pertinent initial phenotype features and length of follow-up for the treatment groups. We selected the 6- and 12-month follow-up time node to evaluate and compare the volume changes of nodules and it resulted in similar consequence. Except for the ablation techniques [52], Huh et al and Døssing et al [38,43] also demonstrated that multiple-session ablation was superior to single-session ablation but has no superior advantages on symptomatic and cosmetic problems, which is coincides with our research. Additionally, in our study, three RFA session, using the moving-shot technique and straight internally cooled electrodes, achieves lager volume reduction and seems to have higher probability of being the most efficacious treatment when compared to three sessions of LA or three sessions of EA.

In principle, RFA generated high-frequency oscillating current, and the polarized molecules and ions in the surrounding tissue were vibrated and rubbed by the exposed electrode needle, which was then converted into heat energy. The heat was gradually transmitted to the periphery, causing irreversible thermal coagulation and necrosis of local tissue to kill tissue cells [53,54].

Initial LA studies placed the needle/fiber in the center of the nodule during the ablation [55]. Such a placement would not achieve treatment of the nodule periphery and would therefore lead to regrowth of the nodule margins, as seen with long-term follow-up [56], but LA had more effect on the improvement of symptomatic and cosmetic problems. HIFU ablation could be considered a truly minimally invasive procedure because it is able to induce irreversible tissue necrosis via thermal ablation beneath the skin without skin puncture or incision [57,58], however, the technology for HIFU was not yet mature, it still needed high-quality randomized controlled trials to verify its efficacy. EA belonged to the chemical ablation therapy, the mechanism of ethanol sclerotherapy was that ethanol induces cellular dehydration and protein denaturation, which were followed by coagulation necrosis, reactive fibrosis, and small-vessel thrombosis [59]. Microwaves produced thermal energy by stimulating water molecules in the ablated tissue to oscillate during ablation [60,61]. It heated tissue to cytotoxic levels through which cellular death is caused, afterwards the created coagulative necrosis was degraded by the patients’ own immune system, but it was reported to be less efficient for the complex benign thyroid nodules [62,63]. However, in our analysis, there was no RCT about MWA meeting the inclusion criteria, so more RCTs with higher quality were needed in the future. Additionally, the efficacy of thermal ablation might also rely on differences in type of energy, treatment time and improvement of treatment technique. It was, therefore, unclarified whether these modalities differ in efficacy and overall superiority if factors such as energy delivered per milliliter of thyroid tissue, treatment time, and treatment technique were congruent. More fair comparisons would need to be carried out based on a prospective randomized study to show the difference between these ablation therapies.

Except for nodule volume, symptom score, cosmetic score and complications are also important evaluation index for safety of ablation. In our study, all of ablation therapies performed well for decreasing of symptom score but has no significant advantage in cosmetic score. Additionally, all of the ablation therapies showed no differences in our study in terms of overall complications. The most common complications for thermal ablations were pains and heat sensation but most of the syndrome could recover in 1 or 3 months, few patients would have voice change due to thermal injury and hemorrhage [48,64–66]. As for chemical ablations, transient voice change might be the most common complication owing to injury of the recurrent laryngeal nerve by the leakage of ethanol outside the thyroid gland [33,35,67]. However, most of the studies considered ablation therapies as lower incidence of complications and most of patients could recover in a short term.

This present study was acknowledged to have several limitations. Firstly, in this Bayesian network meta-analysis, some controversy exists related to the difficulty in evaluating the underlying assumptions of exchangeability, consistency, and homogeneity. As presented in **Table 2**, this study and patient characteristics were relatively homogenous, as also shown statistically by the *I2* value in pooled estimates of the percentage mean volume change in each group. Importantly, in most studies, treatment interventions were randomly assigned. Secondly, there were relatively imbalanced number of studies included in this review such as MWA and HIFU, some studies have been excluded to obtain a more homogeneous group of studies allowing comparisons. Therefore, the risk of random errors and a potential publication bias might also exist. But in this study we used Begg’s method to evaluate the bias and did not find any statistical significance in the assessment of publication bias, with symmetrical distribution as shown in the funnel plot, argues against such bias. Moreover, symmetrical distribution as shown in the funnel plot also argues against such bias. Thirdly, none of the trials were double-blinded, because the nature of these techniques is not possible. However, we have evaluated the quality of each passages and find it unlikely to be affected by the lack of blinding. Despite all of the above limitations, this meta-analysis constitutes the best available evidence for percutaneous ablation treatment efficacy of benign thyroid nodules.

## Conclusion

Based on the limited data, RFA, LA and HIFU treatment offer a significant reduction in nodule volume for benign thyroid nodules. RFA appears to be superior to other US-guided percutaneous ablation in reducing benign thyroid nodule volume during short- and long-term follow-up. In terms of symptom and cosmetic problems, EA and RFA had better efficacy than other US-guided percutaneous ablations and there was no significant difference in the incidence of complications. In the subgroup analysis, RFA with 2 treatment sessions showed the most significant effectiveness and EA showed more effectiveness to decrease the volume of cyst benign thyroid nodules during 6-month follow-up. RFA appears to be superior to other treatment in reducing benign thyroid nodule volume and overall complications for wider applications. Further randomized prospective studies focusing on efficacy, side effects, costs, and quality of life in different percutaneous ablation were warranted.

## Supporting information

S1 Checklist PRISMA NMA checklist of items to include when reporting a systematic review involving a network meta-analysis. (DOCX)

S1 Fig Risks of bias in the trials included in the meta-analysis. -, low risk of bias; +, high risk of bias;?, unclear risk of bias.
(DOCX)

S2 Fig The estimated pair-wise summary effects of outcomes that show the 95% CI and CrI of the estimates and the GRADE score. A GRADE score was assessed in each comparison. High inconsistency^1^, high indirectness^2^, high imprecision^3^ (wide CI). (A) Percentage mean change in benign thyroid nodule volume during 6-month follow-up; (B) Percentage mean change in benign thyroid nodule volume during 12-month follow-up; (C) Symptom score change; (D) Cosmetic score change; (E) Overall complication; (F) Percentage mean volume change of solid or predominantly solid thyroid nodule; (G) Percentage mean volume change of cyst or predominantly cyst thyroid nodule. **RFA**: Radiofrequency Ablation with single treatment session**; RFA2**: Radiofrequency Ablation with 2 treatment session**; EA:** ethanol ablation with single treatment session**; EA3**: ethanol ablation with 3 treatment session; **LA**: Laser Ablation with single treatment session**; LA3**: Laser Ablation with 3 treatment session. **HIFU**: High-Intensity Focused Ultrasound.
(DOCX)

S3 Fig Small-study effects assessed via comparison-adjusted network funnel plots. (A) Funnel plot for risk of publication bias of percentage mean change in benign thyroid nodule volume during 6-month follow-up in network meta-analysis. (**A** = Control group**; B** = High-Intensity Focused Ultrasound Ablation**; C** = Laser ablation with single treatment session**; D** = Laser ablation with 3 treatment session**. E** = Ethanol Ablation with single treatment session**. F** = Ethanol Ablation with 3 treatment session**; G** = Radiofrequency Ablation with single treatment session**; H =** Radiofrequency Ablation with 2 treatment session). (B) Funnel plot for risk of publication bias of percentage mean change in benign thyroid nodule volume during 12-month follow-up in network meta-analysis. (**A** = Control group**; B** = Laser ablation with single treatment session**; C** = Radiofrequency Ablation with single treatment session**; D** = Ethanol Ablation with single treatment session). (C) Funnel plot for risk of publication bias of **Symptom Score Change** in network meta-analysis. (**A** = Control group**; B** = Radiofrequency Ablation with single treatment session**; C** = Radiofrequency Ablation with 2 treatment session**; D =** Ethanol Ablation with single treatment session**; E** = Ethanol Ablation with 3 treatment session; **F** = Laser ablation with single treatment session**; G** = Laser ablation with 3 treatment session). (D) Funnel plot for risk of publication bias of cosmetic score change in network meta-analysis. (**A** = Control group**; B** = Radiofrequency Ablation with single treatment session**; C** = Radiofrequency Ablation with 2 treatment session**; D =** Ethanol Ablation with single treatment session**; E** = Ethanol Ablation with 3 treatment session; **F** = Laser ablation with single treatment session**; G** = Laser ablation with 3 treatment session). **(E)** Funnel plot for risk of publication bias of percentage mean change of solid or predominantly solid thyroid nodule volume in network meta-analysis. (**A** = Control group**; B** = Radiofrequency Ablation with single treatment session**; C** = Radiofrequency Ablation with 2 treatment session**; D =** Ethanol Ablation with single treatment session**; E** = Ethanol Ablation with 3 treatment session; **F** = Laser ablation with single treatment session**; G** = Laser ablation with 3 treatment session**; H =** High-Intensity Focused Ultrasound Ablation). (F) Funnel plot for risk of publication bias of percentage mean change of cyst or predominantly cyst thyroid nodule volume in network meta-analysis. (**A** = Control group**; B** = Radiofrequency Ablation with single treatment session**; C** = Ethanol Ablation with single treatment session**; D** = Laser ablation with single treatment session). (G) Funnel plot for risk of publication bias of overall complication in network meta-analysis. (**A** = Control group**; B** = Radiofrequency Ablation with single treatment session**; C** = Radiofrequency Ablation with 2 treatment session**; D =** Ethanol Ablation with single treatment session**; E** = Ethanol Ablation with 3 treatment session; **F** = Laser ablation with single treatment session**; G** = Laser ablation with 3 treatment session**; H =** High-Intensity Focused Ultrasound Ablation).
(DOCX)

S4 Fig **A.** Evaluation of the local inconsistency by loop specific approach for percentage mean change in benign thyroid nodules after percutaneous ablation therapies. **B.** Evaluation of the local inconsistency by loop specific approach for overall complication in benign thyroid nodules.
(DOCX)

S5 Fig The cumulative ranking curve of the outcomes of the different results. The surface under the cumulative ranking curve (SUCRA) represents the ranking of devices. A higher SUCRA suggests a higher probability of being good treatment. (A) Percentage mean change in benign thyroid nodule volume during 6-month follow-up; (B) Percentage mean change in benign thyroid nodule volume during 12-month follow-up; (C) Symptom score change; (D) Cosmetic score change; (E) Overall complication; (F) Percentage mean volume change of solid or predominantly solid thyroid nodule; (G) Percentage mean volume change of cyst or predominantly cyst thyroid nodule. **RFA**: Radiofrequency Ablation with single treatment session**; RFA2**: Radiofrequency Ablation with 2 treatment session**; EA:** ethanol ablation with single treatment session**; EA3**: ethanol ablation with 3 treatment session; **LA**: Laser Ablation with single treatment session**; LA3**: Laser Ablation with 3 treatment session. **HIFU**: High-Intensity Focused Ultrasound.
(DOCX)

S6 Fig Contribution matrix of the network meta-analysis. Numbers represent percentage contribution of each direct comparison to the network meta-analysis estimate of each comparison. Direct comparisons are represented in the columns of the matrix. Network estimates are represented in the rows of the matrix. Each direct comparison in network meta-analysis contributes differently to the estimation of the network summary effects. The matrix is useful to identify the most influential comparisons for each network estimate and for the entire network. The weight of each direct comparison is a combination of the variance of the direct treatment effect and the network structure. **(A)** Contribution matrix of percentage mean change during 6-month follow-up in the network meta-analysis (**A** = Control group; **B** = High-Intensity Focused Ultrasound Ablation; **C** = Laser ablation with single treatment session**; D** = Laser ablation with 3 treatment session; **E** = Ethanol Ablation with single treatment session; **F =** Ethanol Ablation with 3 treatment session; **G** = Radiofrequency Ablation with single treatment session; **H =** Radiofrequency Ablation with 2 treatment session**). (B)** Contribution matrix of percentage mean change during 12-month follow-up in the network meta-analysis (**A** = Control group; **B** = Laser ablation with single treatment session**; C** = Radiofrequency Ablation with single treatment session; **D** = Ethanol Ablation with single treatment session**). (C)** Contribution matrix of symptom score change in the network meta-analysis (**A** = Control group**; B** = Radiofrequency Ablation with single treatment session**; C** = Radiofrequency Ablation with 2 treatment session**; D =** Ethanol Ablation with single treatment session**; E** = Ethanol Ablation with 3 treatment session; **F** = Laser ablation with single treatment session**; G** = Laser ablation with 3 treatment session**). (D)** Contribution matrix of cosmetic score change in the network meta-analysis (**A** = Control group**; B** = Radiofrequency Ablation with single treatment session**; C** = Radiofrequency Ablation with 2 treatment session**; D =** Ethanol Ablation with single treatment session**; E** = Ethanol Ablation with 3 treatment session; **F** = Laser ablation with single treatment session**; G** = Laser ablation with 3 treatment session**). (E)** Contribution matrix of overall complication in the network meta-analysis (**A** = Control group**; B** = Radiofrequency Ablation with single treatment session**; C** = Radiofrequency Ablation with 2 treatment session**; D =** Ethanol Ablation with single treatment session**; E** = Ethanol Ablation with 3 treatment session; **F** = Laser ablation with single treatment session**; G** = Laser ablation with 3 treatment session**; H =** High-Intensity Focused Ultrasound Ablation**). (F)** Contribution matrix of percentage mean change of solid or predominantly solid thyroid nodule volume in the network meta-analysis (**A** = Control group**; B** = Radiofrequency Ablation with single treatment session**; C** = Radiofrequency Ablation with 2 treatment session**; D =** Ethanol Ablation with single treatment session**; E** = Ethanol Ablation with 3 treatment session; **F** = Laser ablation with single treatment session**; G** = Laser ablation with 3 treatment session**). (G)** Contribution matrix of percentage mean change of cyst or predominantly cyst thyroid nodule volume in the network meta-analysis (**A** = Control group; **B** = Ethanol Ablation with single treatment session**; C** = Radiofrequency Ablation with single treatment session; **D** = Laser ablation with single treatment session**).**
(DOCX)

S1 Table Study strategy. (DOCX)

S2 Table Summary of the complications of the included studies. (DOCX)

S3 Table Results of network meta-analysis for overall complication in benign thyroid nodules after percutaneous ablation therapies. Odds ratios are presented in the cells in common between the column-defining and row-defining agents. [OR: odds ratio; 95%CrI: 95% Credible Intervals].
(DOCX)

## Abbreviations

RFA: Radiofrequency Ablation
EA: Ethanol ablation
LA: Laser Ablation
HIFU: high-intensity focused ultrasound
RCT: Randomized Controlled Trial
SUCRA: surface under the cumulative ranking curve
