# Supplementary material for: Comparative efficacy of different ultrasound-guided ablation for the treatment of benign thyroid nodules: Systematic review and network meta-analysis of randomized controlled trials
Source: PLoS One. 2021 Jan 20;16(1):e0243864. doi: 10.1371/journal.pone.0243864 (PMC7816973; doi:10.1371/journal.pone.0243864)

**Supplementary Figure 1**

Risks of bias in the trials included in the meta-analysis. -, low risk of bias; +, high risk of bias; ?, unclear risk of bias.


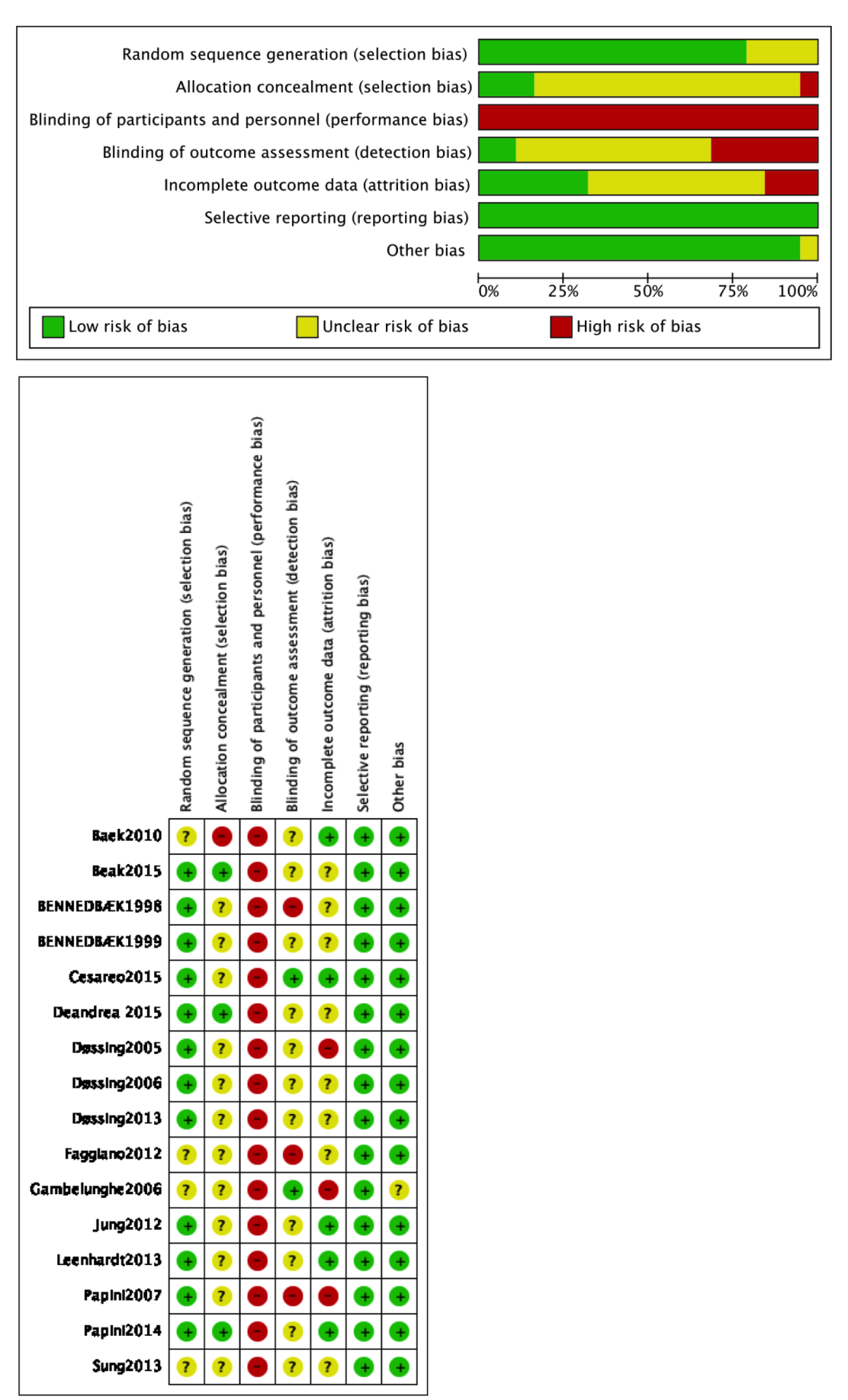

Supplement: S1 Fig — -, low risk of bias; +, high risk of bias;?, unclear risk of bias. (DOCX) [file pone.0243864.s002.docx]
