# Supplementary material for: Comparative efficacy of different ultrasound-guided ablation for the treatment of benign thyroid nodules: Systematic review and network meta-analysis of randomized controlled trials
Source: PLoS One. 2021 Jan 20;16(1):e0243864. doi: 10.1371/journal.pone.0243864 (PMC7816973; doi:10.1371/journal.pone.0243864)

**Supplementary Figure 3**

Small-study effects assessed via comparison-adjusted network funnel plots.

(A) Funnel plot for risk of publication bias of percentage mean change in benign thyroid nodule volume during 6-month follow-up in network meta-analysis. (**A**=Control group**; B**= High-Intensity Focused Ultrasound Ablation**; C**=Laser ablation with single treatment session**; D**=Laser ablation with 3 treatment session**. E**=Ethanol Ablation with single treatment session**. F**=Ethanol Ablation with 3 treatment session**; G**=Radiofrequency Ablation with single treatment session**; H=**Radiofrequency Ablation with 2 treatment session)


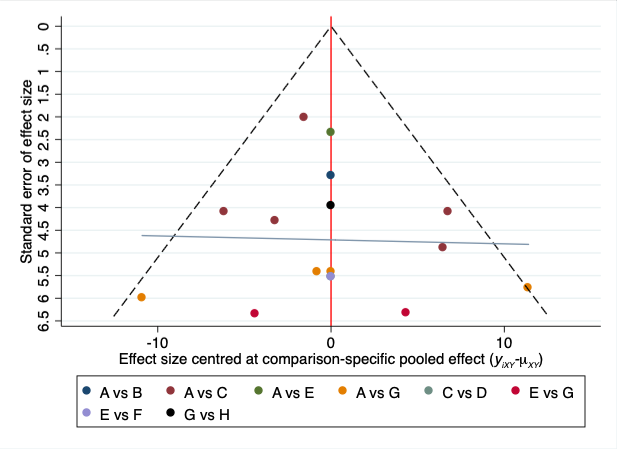


(B) Funnel plot for risk of publication bias of percentage mean change in benign thyroid nodule volume during 12-month follow-up in network meta-analysis. (**A**=Control group**; B**= Laser ablation with single treatment session**; C**= Radiofrequency Ablation with single treatment session**; D**=Ethanol Ablation with single treatment session**.**)

**
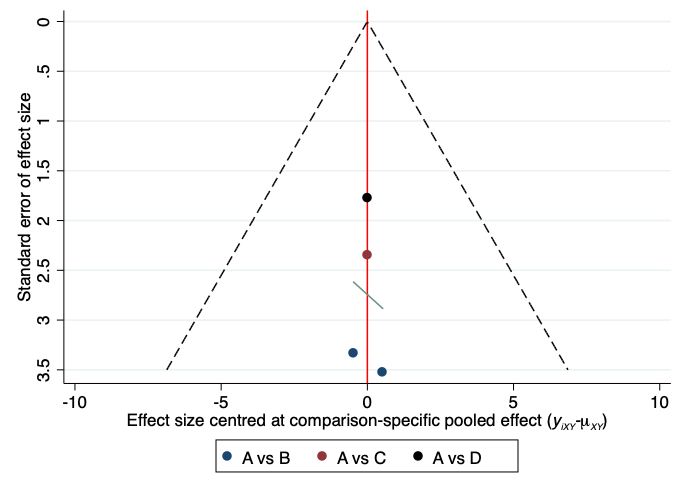
**

(C) Funnel plot for risk of publication bias of **Symptom Score Change** in network meta-analysis.（**A**=Control group**; B**=Radiofrequency Ablation with single treatment session**; C**=Radiofrequency Ablation with 2 treatment session**; D=** Ethanol Ablation with single treatment session**; E**= Ethanol Ablation with 3 treatment session; **F**=Laser ablation with single treatment session**; G**= Laser ablation with 3 treatment session）

1. **
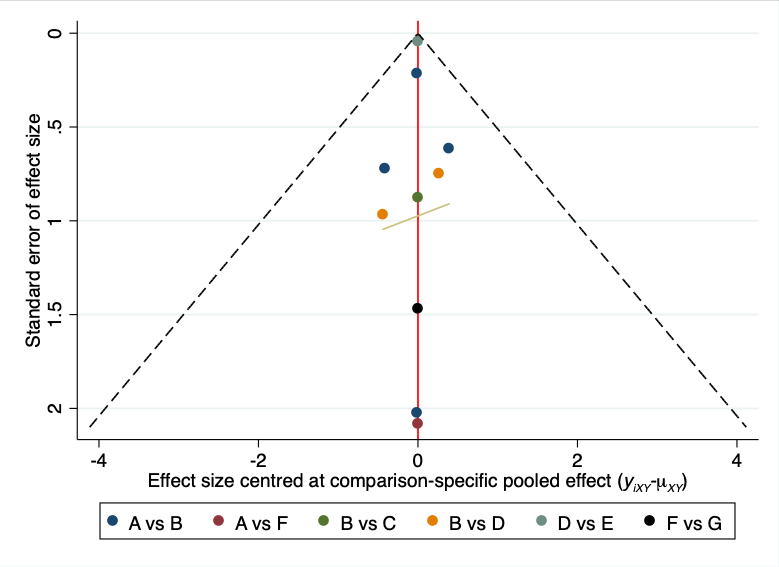
**

(D) Funnel plot for risk of publication bias of cosmetic score change in network meta-analysis.(**A**=Control group**; B**=Radiofrequency Ablation with single treatment session**; C**=Radiofrequency Ablation with 2 treatment session**; D=** Ethanol Ablation with single treatment session**; E**= Ethanol Ablation with 3 treatment session; **F**=Laser ablation with single treatment session**; G**= Laser ablation with 3 treatment session)


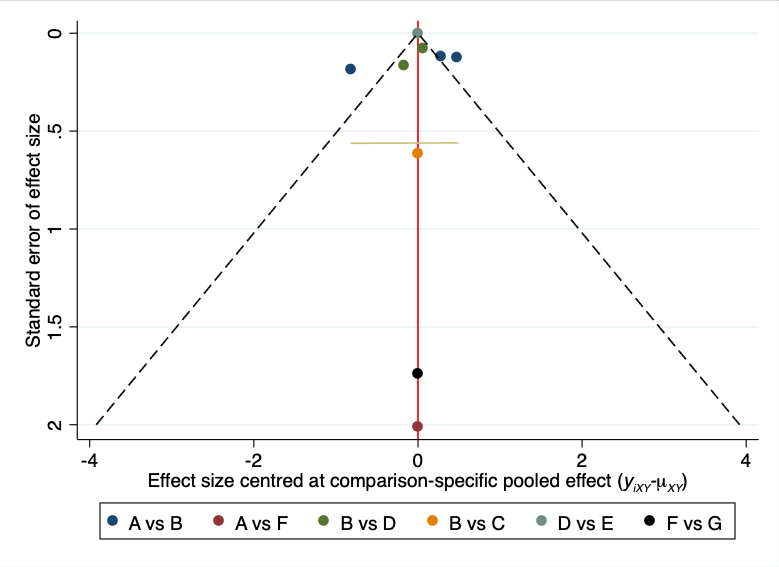


**(E)** Funnel plot for risk of publication bias of percentage mean change of solid or predominantly solid thyroid nodule volume in network meta-analysis. (**A**=Control group**; B**=Radiofrequency Ablation with single treatment session**; C**=Radiofrequency Ablation with 2 treatment session**; D=** Ethanol Ablation with single treatment session**; E**= Ethanol Ablation with 3 treatment session; **F**=Laser ablation with single treatment session**; G**= Laser ablation with 3 treatment session**; H=**High-Intensity Focused Ultrasound Ablation)


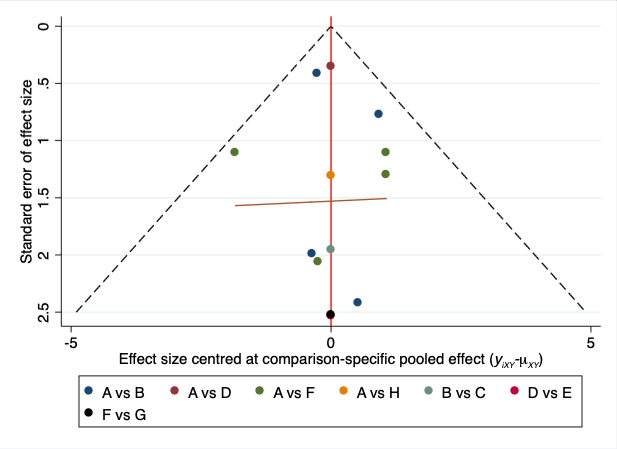


(F) Funnel plot for risk of publication bias of percentage mean change of cyst or predominantly cyst thyroid nodule volume in network meta-analysis. (**A**=Control group**; B**=Radiofrequency Ablation with single treatment session**; C**= Ethanol Ablation with single treatment session**; D**=Laser ablation with single treatment session)


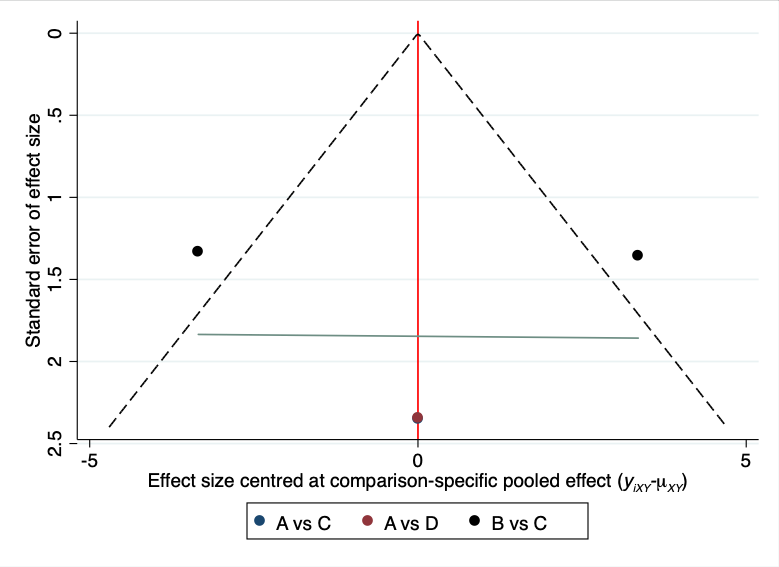


(G) Funnel plot for risk of publication bias of overall complication in network meta-analysis. (**A**=Control group**; B**=Radiofrequency Ablation with single treatment session**; C**=Radiofrequency Ablation with 2 treatment session**; D=** Ethanol Ablation with single treatment session**; E**= Ethanol Ablation with 3 treatment session; **F**=Laser ablation with single treatment session**; G**= Laser ablation with 3 treatment session**; H=**High-Intensity Focused Ultrasound Ablation)


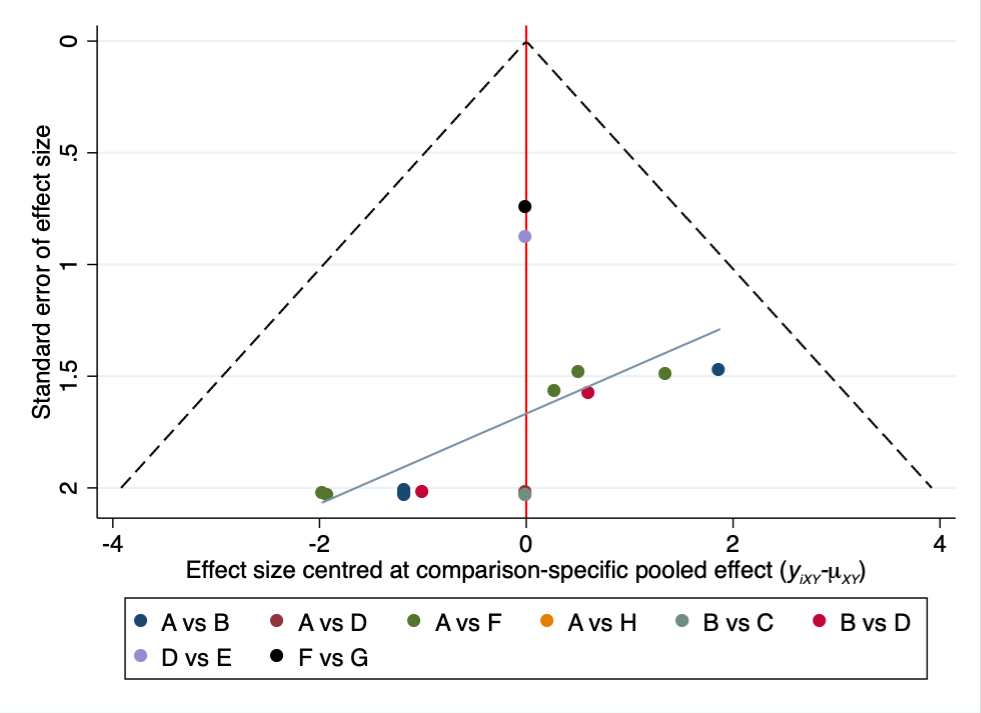

Supplement: S3 Fig — (A) Funnel plot for risk of publication bias of percentage mean change in benign thyroid nodule volume during 6-month follow-up in network meta-analysis. (A = Control group; B = High-Intensity Focused Ultrasound Ablation; C = Laser ablation with single treatment session; D = Laser ablation with 3 treatment session. E = Ethanol Ablation with single treatment session. F = Ethanol Ablation with 3 treatment session; G = Radiofrequency Ablation with single treatment session; H = Radiofrequency Ablation with 2 treatment session). (B) Funnel plot for risk of publication bias of percentage mean change in benign thyroid nodule volume during 12-month follow-up in network meta-analysis. (A = Control group; B = Laser ablation with single treatment session; C = Radiofrequency Ablation with single treatment session; D = Ethanol Ablation with single treatment session). (C) Funnel plot for risk of publication bias of Symptom Score Change in network meta-analysis. (A = Control group; B = Radiofrequency Ablation with single treatment session; C = Radiofrequency Ablation with 2 treatment session; D = Ethanol Ablation with single treatment session; E = Ethanol Ablation with 3 treatment session; F = Laser ablation with single treatment session; G = Laser ablation with 3 treatment session). (D) Funnel plot for risk of publication bias of cosmetic score change in network meta-analysis. (A = Control group; B = Radiofrequency Ablation with single treatment session; C = Radiofrequency Ablation with 2 treatment session; D = Ethanol Ablation with single treatment session; E = Ethanol Ablation with 3 treatment session; F = Laser ablation with single treatment session; G = Laser ablation with 3 treatment session). (E) Funnel plot for risk of publication bias of percentage mean change of solid or predominantly solid thyroid nodule volume in network meta-analysis. (A = Control group; B = Radiofrequency Ablation with single treatment session; C = Radiofrequency Ablation with 2 treatment session; D [file pone.0243864.s004.docx]
