# Supplementary material for: Comparative efficacy of different ultrasound-guided ablation for the treatment of benign thyroid nodules: Systematic review and network meta-analysis of randomized controlled trials
Source: PLoS One. 2021 Jan 20;16(1):e0243864. doi: 10.1371/journal.pone.0243864 (PMC7816973; doi:10.1371/journal.pone.0243864)
Supplement: S4 Fig — A. Evaluation of the local inconsistency by loop specific approach for percentage mean change in benign thyroid nodules after percutaneous ablation therapies. B. Evaluation of the local inconsistency by loop specific approach for overall complication in benign thyroid nodules. (DOCX) [file pone.0243864.s005.docx]

**Supplementary Figure 4A.** Evaluation of the local inconsistency by loop specific approach for percentage mean change in benign thyroid nodules after percutaneous ablation therapies.**
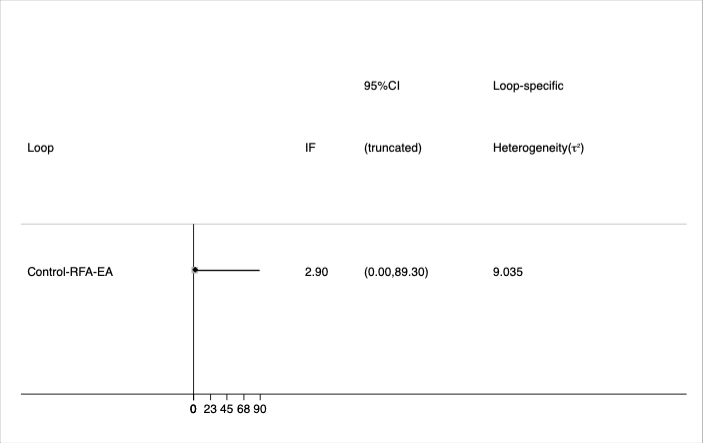
**

**
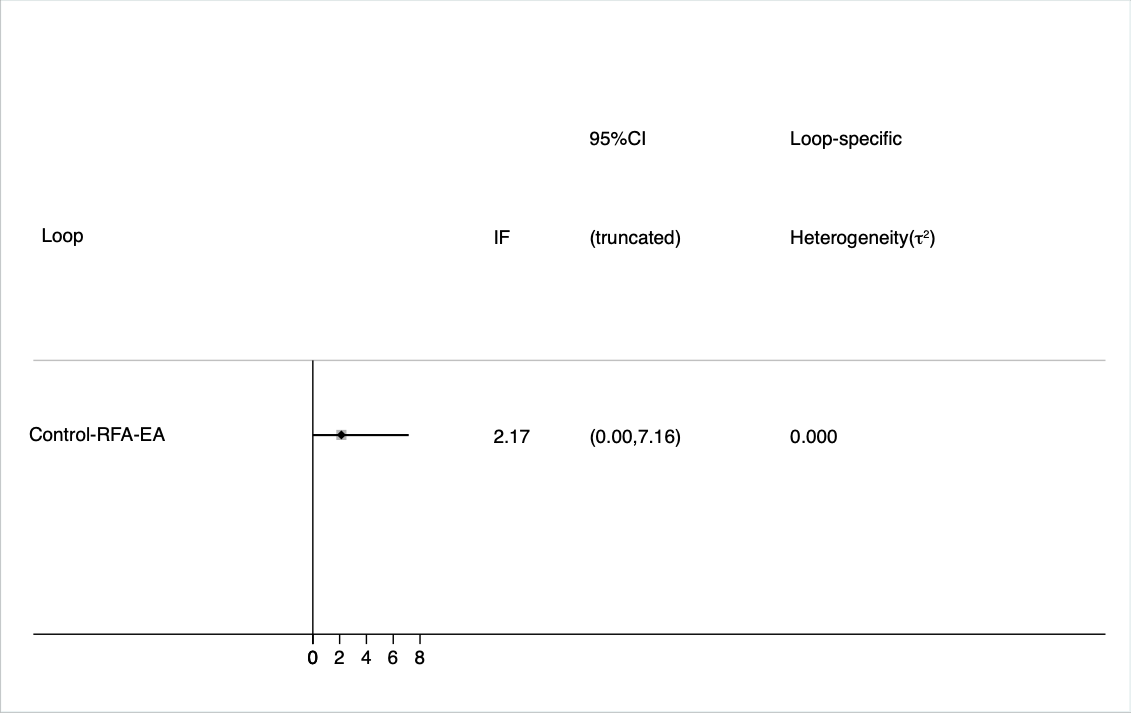
Supplementary Figure 4B.** Evaluation of the local inconsistency by loop specific approach for overall complication in benign thyroid nodules
