# Supplementary material for: Comparative efficacy of different ultrasound-guided ablation for the treatment of benign thyroid nodules: Systematic review and network meta-analysis of randomized controlled trials
Source: PLoS One. 2021 Jan 20;16(1):e0243864. doi: 10.1371/journal.pone.0243864 (PMC7816973; doi:10.1371/journal.pone.0243864)
Supplement: S5 Fig — The surface under the cumulative ranking curve (SUCRA) represents the ranking of devices. A higher SUCRA suggests a higher probability of being good treatment. (A) Percentage mean change in benign thyroid nodule volume during 6-month follow-up; (B) Percentage mean change in benign thyroid nodule volume during 12-month follow-up; (C) Symptom score change; (D) Cosmetic score change; (E) Overall complication; (F) Percentage mean volume change of solid or predominantly solid thyroid nodule; (G) Percentage mean volume change of cyst or predominantly cyst thyroid nodule. RFA: Radiofrequency Ablation with single treatment session; RFA2: Radiofrequency Ablation with 2 treatment session; EA: ethanol ablation with single treatment session; EA3: ethanol ablation with 3 treatment session; LA: Laser Ablation with single treatment session; LA3: Laser Ablation with 3 treatment session. HIFU: High-Intensity Focused Ultrasound. (DOCX) [file pone.0243864.s006.docx]

**Supplementary Figure 5**

**The cumulative ranking curve of the outcomes of the different results.** The surface under the cumulative ranking curve (SUCRA) represents the ranking of devices. A higher SUCRA suggests a higher probability of being good treatment. (A) Percentage mean change in benign thyroid nodule volume during 6-month follow-up; (B) Percentage mean change in benign thyroid nodule volume during 12-month follow-up; (C) Symptom score change; (D) Cosmetic score change; (E) Overall complication; (F) Percentage mean volume change of solid or predominantly solid thyroid nodule; (G) Percentage mean volume change of cyst or predominantly cyst thyroid nodule. **RFA**: Radiofrequency Ablation with single treatment session**; RFA2**: Radiofrequency Ablation with 2 treatment session**; EA:** ethanol ablation with single treatment session**; EA3**: ethanol ablation with 3 treatment session; **LA**: Laser Ablation with single treatment session**; LA3**: Laser Ablation with 3 treatment session. **HIFU**: High-Intensity Focused Ultrasound.

**
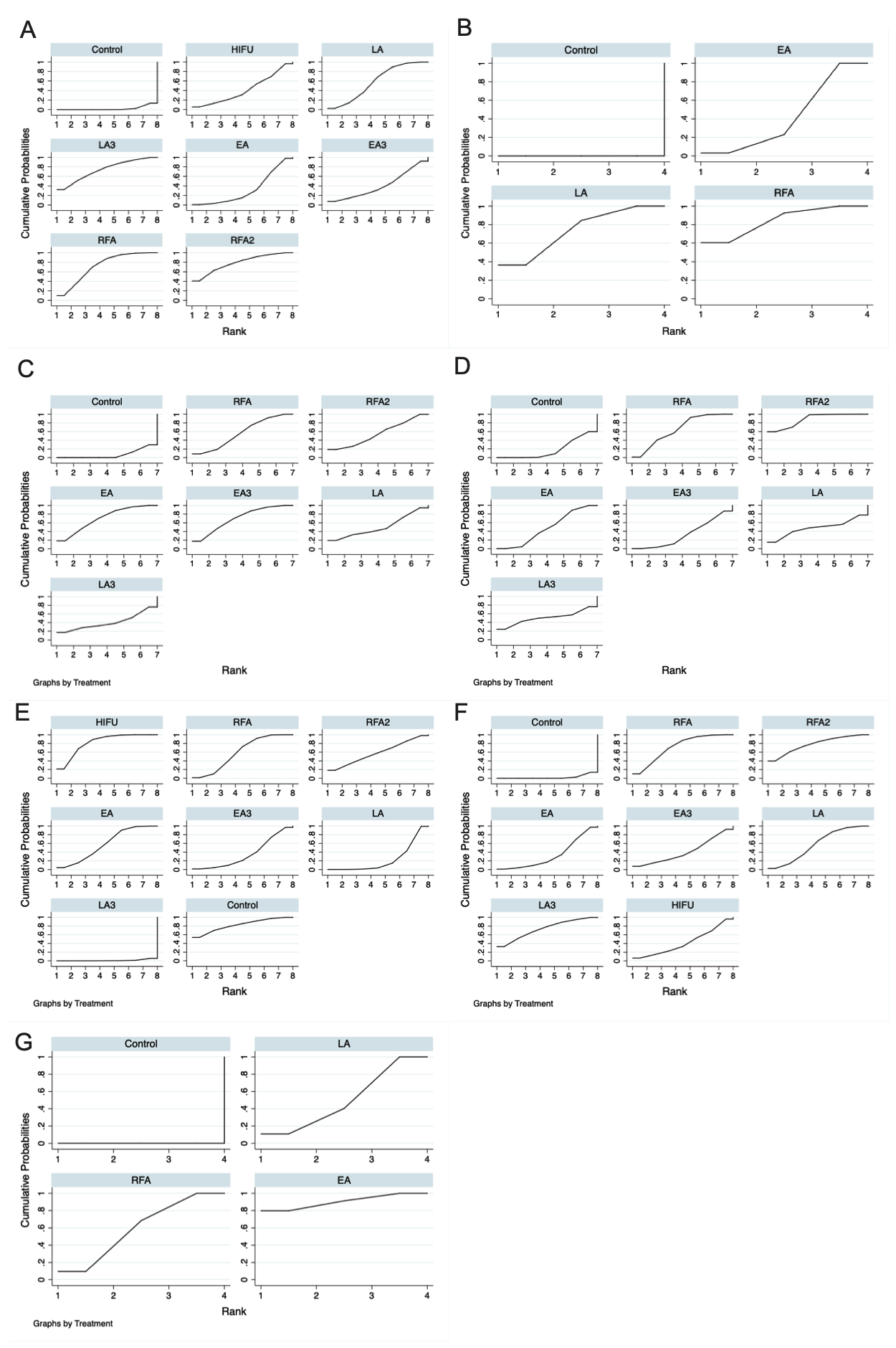
**
