# Supplementary material for: Comparative efficacy of different ultrasound-guided ablation for the treatment of benign thyroid nodules: Systematic review and network meta-analysis of randomized controlled trials
Source: PLoS One. 2021 Jan 20;16(1):e0243864. doi: 10.1371/journal.pone.0243864 (PMC7816973; doi:10.1371/journal.pone.0243864)

**Supplementary Figure 6**

Contribution matrix of the network meta-analysis. Numbers represent percentage contribution of each direct comparison to the network meta-analysis estimate of each comparison. Direct comparisons are represented in the columns of the matrix. Network estimates are represented in the rows of the matrix. Each direct comparison in network meta-analysis contributes differently to the estimation of the network summary effects. The matrix is useful to identify the most influential comparisons for each network estimate and for the entire network. The weight of each direct comparison is a combination of the variance of the direct treatment effect and the network structure.

**(A)** Contribution matrix of percentage mean change during 6-month follow-up in the network meta-analysis (**A**=Control group; **B** =High-Intensity Focused Ultrasound Ablation; **C**=Laser ablation with single treatment session**; D**=Laser ablation with 3 treatment session; **E**=Ethanol Ablation with single treatment session; **F=**Ethanol Ablation with 3 treatment session; **G**=Radiofrequency Ablation with single treatment session; **H=**Radiofrequency Ablation with 2 treatment session**)**


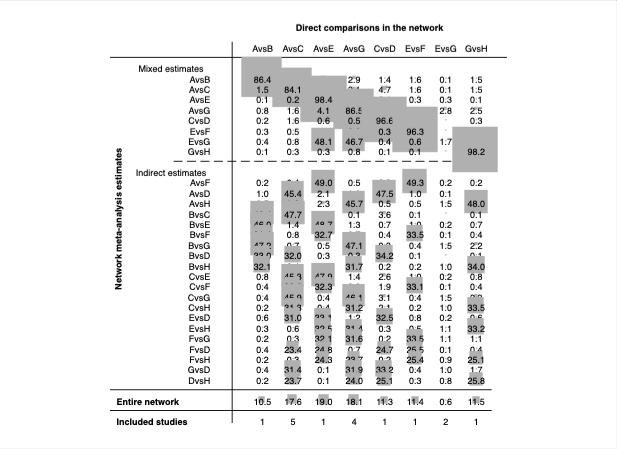


**(B)** Contribution matrix of percentage mean change during 12-month follow-up in the network meta-analysis (**A**=Control group; **B**=Laser ablation with single treatment session**; C**=Radiofrequency Ablation with single treatment session; **D**=Ethanol Ablation with single treatment session**)**


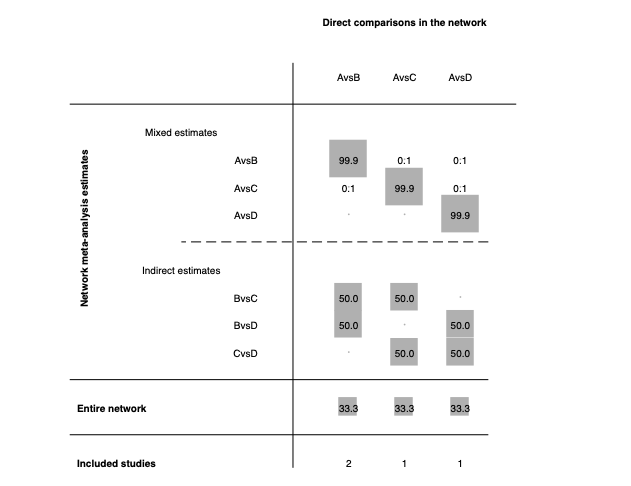


**(C)** Contribution matrix of symptom score change in the network meta-analysis (**A**=Control group**; B**=Radiofrequency Ablation with single treatment session**; C**=Radiofrequency Ablation with 2 treatment session**; D=** Ethanol Ablation with single treatment session**; E**= Ethanol Ablation with 3 treatment session; **F**=Laser ablation with single treatment session**; G**= Laser ablation with 3 treatment session**)**


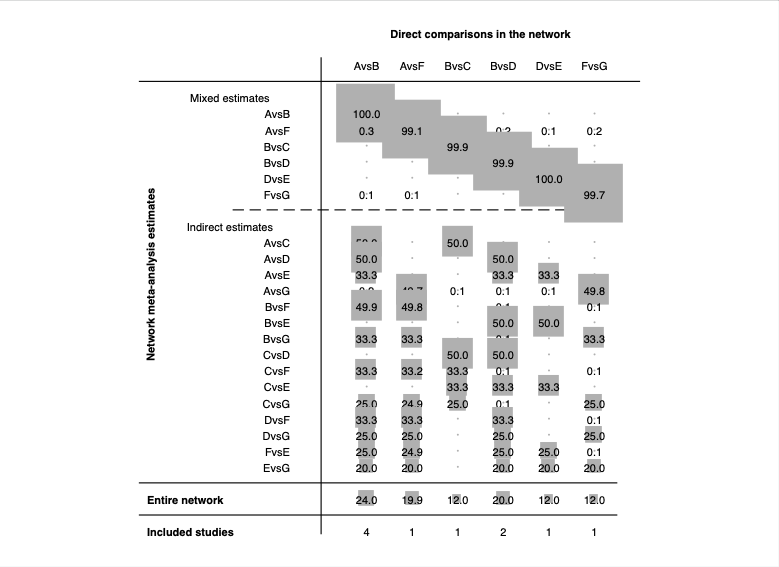


**(D)** Contribution matrix of cosmetic score change in the network meta-analysis (**A**=Control group**; B**=Radiofrequency Ablation with single treatment session**; C**=Radiofrequency Ablation with 2 treatment session**; D=** Ethanol Ablation with single treatment session**; E**= Ethanol Ablation with 3 treatment session; **F**=Laser ablation with single treatment session**; G**= Laser ablation with 3 treatment session**)**


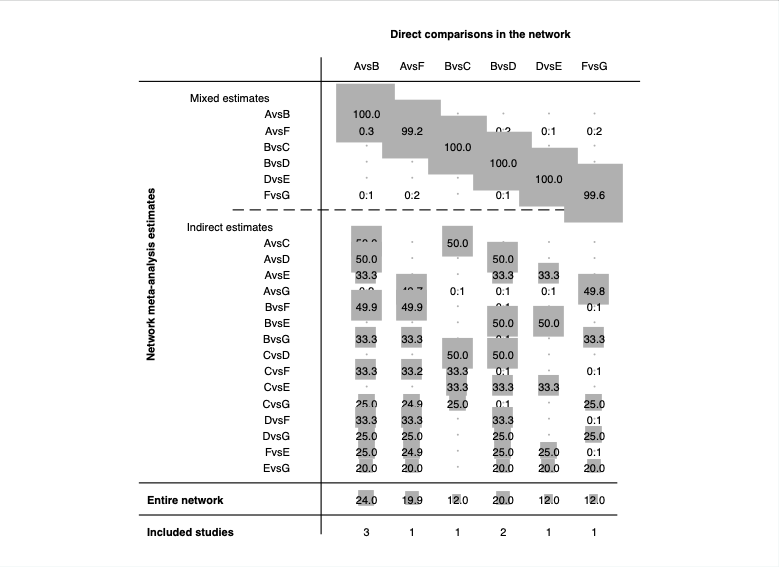


**(E)** Contribution matrix of overall complication in the network meta-analysis (**A**=Control group**; B**=Radiofrequency Ablation with single treatment session**; C**=Radiofrequency Ablation with 2 treatment session**; D=** Ethanol Ablation with single treatment session**; E**= Ethanol Ablation with 3 treatment session; **F**=Laser ablation with single treatment session**; G**= Laser ablation with 3 treatment session**; H=**High-Intensity Focused Ultrasound Ablation**)**


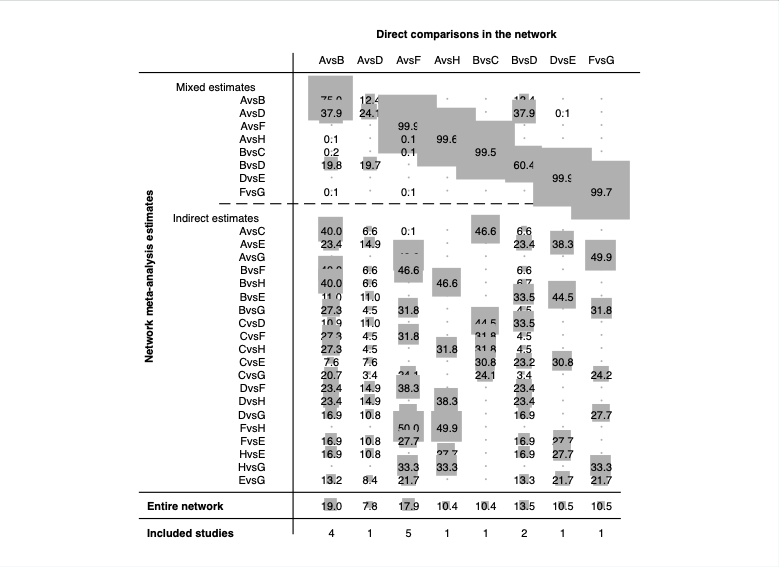


**(F)** Contribution matrix of percentage mean change of solid or predominantly solid thyroid nodule volume in the network meta-analysis (**A**=Control group**; B**=Radiofrequency Ablation with single treatment session**; C**=Radiofrequency Ablation with 2 treatment session**; D=** Ethanol Ablation with single treatment session**; E**= Ethanol Ablation with 3 treatment session; **F**=Laser ablation with single treatment session**; G**= Laser ablation with 3 treatment session**)**


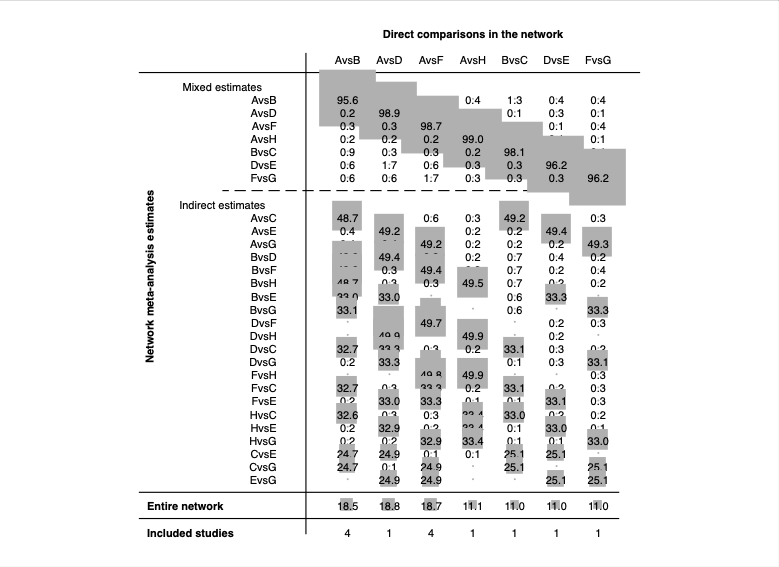


**(G)** Contribution matrix of percentage mean change of cyst or predominantly cyst thyroid nodule volume in the network meta-analysis (**A**=Control group; **B**=Ethanol Ablation with single treatment session**; C**=Radiofrequency Ablation with single treatment session; **D**= Laser ablation with single treatment session**)**


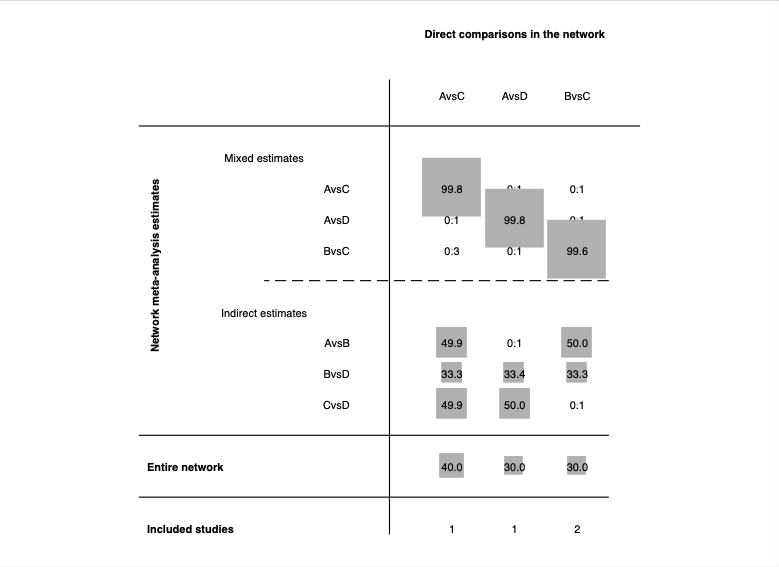

Supplement: S6 Fig — Numbers represent percentage contribution of each direct comparison to the network meta-analysis estimate of each comparison. Direct comparisons are represented in the columns of the matrix. Network estimates are represented in the rows of the matrix. Each direct comparison in network meta-analysis contributes differently to the estimation of the network summary effects. The matrix is useful to identify the most influential comparisons for each network estimate and for the entire network. The weight of each direct comparison is a combination of the variance of the direct treatment effect and the network structure. (A) Contribution matrix of percentage mean change during 6-month follow-up in the network meta-analysis (A = Control group; B = High-Intensity Focused Ultrasound Ablation; C = Laser ablation with single treatment session; D = Laser ablation with 3 treatment session; E = Ethanol Ablation with single treatment session; F = Ethanol Ablation with 3 treatment session; G = Radiofrequency Ablation with single treatment session; H = Radiofrequency Ablation with 2 treatment session). (B) Contribution matrix of percentage mean change during 12-month follow-up in the network meta-analysis (A = Control group; B = Laser ablation with single treatment session; C = Radiofrequency Ablation with single treatment session; D = Ethanol Ablation with single treatment session). (C) Contribution matrix of symptom score change in the network meta-analysis (A = Control group; B = Radiofrequency Ablation with single treatment session; C = Radiofrequency Ablation with 2 treatment session; D = Ethanol Ablation with single treatment session; E = Ethanol Ablation with 3 treatment session; F = Laser ablation with single treatment session; G = Laser ablation with 3 treatment session). (D) Contribution matrix of cosmetic score change in the network meta-analysis (A = Control group; B = Radiofrequency Ablation with single treatment session; C = Radiofrequency Ablation with 2 treatment sess [file pone.0243864.s007.docx]
