# Supplementary material for: Comparative efficacy of different ultrasound-guided ablation for the treatment of benign thyroid nodules: Systematic review and network meta-analysis of randomized controlled trials
Source: PLoS One. 2021 Jan 20;16(1):e0243864. doi: 10.1371/journal.pone.0243864 (PMC7816973; doi:10.1371/journal.pone.0243864)
Supplement: S1 Table — (DOCX) [file pone.0243864.s008.docx]

**Table S1. Study strategy.**

|  | PubMed | No. | Embase | No. | Cochrane Trials | No. |
| --- | --- | --- | --- | --- | --- | --- |
| #1 | "thyroid nodule"[Title/Abstract] OR "thyroid benign nodule"[Title/Abstract] | 4206 | **' thyroid nodule '/exp OR ' thyroid benign nodule '/exp OR intubat*:ti,ab** | 11256 | **([mh "thyroid nodulel"] OR Intubat*:ti,ab,kw) AND (thyroid benign nodule):ti,ab,kw** | 1036 |
| #2 | ("RCT"[All Fields] OR "randomized controlled trial"[Title/Abstract]) AND ("thyroid nodule"[MeSH Terms] OR ("thyroid"[All Fields] AND "nodule"[All Fields]) OR "thyroid nodule"[All Fields]) | 162 | **RCT/exp OR ( randomized controlled trial/exp AND** thyroid **nodule/exp)** | 103 | ([mh RCT] OR [mh randomized controlled trial]) AND [mh "thyroid nodule"] | 67 |
| #3 | "percutaneous ablation"[Title/Abstract] OR "radiofrequency"[Title/Abstract] OR "ethanol"[Title/Abstract] OR "laser"[Title/Abstract] OR "microwave"[Title/Abstract] OR "high intensity focused ultrasound ablation"[Title/Abstract] | 1689 | **(percutaneous ablation* OR (radiofrequency* OR RFA OR radiofrequency ablation) OR (ethanol OR percutaneous ethanol injection OR EA) OR (laser OR LA OR interstitial photocoagulation) OR ((microwave OR MWA) OR high intensity focused ultrasound):ti,ab** | 525 | (percutaneous ablation* OR (radiofrequency* OR RFA OR radiofrequency ablation) OR (ethanol OR percutaneous ethanol injection OR EA) OR (laser OR LA OR interstitial photocoagulation) OR ((microwave OR MWA) OR high intensity focused ultrasound):ti,ab,kw | 798 |
| #4 | #2 AND #3 | 130 | #2 AND #3 | 46 | #2 AND #3 | 102 |
| #5 | #1 AND #4 | 30 | #1 AND #4 | 21 | #1 AND #4 | 32 |
