# Supplementary material for: Comparative efficacy of different ultrasound-guided ablation for the treatment of benign thyroid nodules: Systematic review and network meta-analysis of randomized controlled trials
Source: PLoS One. 2021 Jan 20;16(1):e0243864. doi: 10.1371/journal.pone.0243864 (PMC7816973; doi:10.1371/journal.pone.0243864)
Supplement: S2 Table — (DOCX) [file pone.0243864.s009.docx]

**Table S2.** Summary of the complications of the included studies

| **First author** | **Year** | **Country** | **Major Complication** | **Major Complication** | **Side Effect** |
| --- | --- | --- | --- | --- | --- |
| Maurilio Deandrea | 2015 | Italy | 0 | 0 | 0 |
| Jung Hwan Baek | 2015 | Korea | 1(transient voice change) | 0 | 0 |
| Roberto Cesareo | 2015 | Italy | 1(permanent right paramedian vocal cord palsy with inspiratory stridor without dysphonia.) | 8(pain), 2(transient voice change） | 0 |
| Enrico Papini | 2014 | Italy | 1(vocal cord paresis) | 4(low-grade fever） | 0 |
| Laurence Leenhardt | 2013 | France | 0 | 0 | 0 |
| Jin Yong Sung | 2013 | Korea | 0 | 0 | 0 |
| Helle Døssing | 2013 | Denmark | 0 | 8(slight to moderate pain) | 11(mild pain） |
| Jung Yin Huh | 2012 | Korea | 0 | 0 | All(mild pain） |
| Faggiano | 2012 | Italy | 0 | 0 | All(heat sensation） |
| Jung Hwan Baek | 2010 | Korea | 0 | 0 | All(heat sensation） |
| Enrico Papini | 2007 | Italy | 0 | 4(mild pain), 8(persistent tachycardia or nervousness) | 0 |
| Gambelunghe | 2006 | Italy | 0 | 3(fever), 8(transient hyperthyroidism) | 5(mild pain or burning） |
| Helle Døssing | 2006 | Denmark | 0 | 7 had moderate pain in ILP-3 group | 6 had moderate pain and 4 had tenderness in ILP-1group and 6 had moderate pain in ILP-3 group |
| Helle Døssing | 2005 | Denmark | 0 | 0 | 3(tenderness） |
| BENNEDBÆK, F. N | 1999 | Denmark | 2(severe pain), 1(carotidynia-like syndrome) | 2(Hyperpyrexia), 1(Transient dysphonia) | 0 |
| BENNEDBÆK, F. N | 1998 | Denmark | 0 | 0 | 0 |
