# Supplementary material for: Comparative efficacy of different ultrasound-guided ablation for the treatment of benign thyroid nodules: Systematic review and network meta-analysis of randomized controlled trials
Source: PLoS One. 2021 Jan 20;16(1):e0243864. doi: 10.1371/journal.pone.0243864 (PMC7816973; doi:10.1371/journal.pone.0243864)
Supplement: S3 Table — Odds ratios are presented in the cells in common between the column-defining and row-defining agents. [OR: odds ratio; 95%CrI: 95% Credible Intervals]. (DOCX) [file pone.0243864.s010.docx]

**Table S3. Results of network meta-analysis for overall complication in benign thyroid nodules after percutaneous ablation therapies.** Odds ratios are presented in the cells in common between the column-defining and row-defining agents. [OR: odds ratio; 95%CrI: 95% Credible Intervals].

| **Control** |  |  |  |  |  |  |  |
| --- | --- | --- | --- | --- | --- | --- | --- |
| 1.87 (0.03,100.53) | **HIFU** |  |  |  |  |  |  |
| 0.41 (0.08,2.18) | 0.22 (0.00,16.51) | **RFA** |  |  |  |  |  |
| 0.41 (0.01,30.81) | 0.22 (0.00,78.21) | 1.00 (0.02,53.66) | **RFA2** |  |  |  |  |
| 0.25 (0.02,2.81) | 0.14 (0.00,14.25) | 0.62 (0.07,5.19) | 0.62 (0.01,56.53) | **EA** |  |  |  |
| 0.15 (0.03,0.63) | 0.08 (0.00,5.46) | 0.36 (0.04,3.29) | 0.36 (0.00,34.14) | 0.58 (0.03,9.61) | **LA** |  |  |
| 0.09 (0.00,1.75) | 0.05 (0.00,6.94) | 0.22 (0.01,3.42) | 0.22 (0.00,27.77) | 0.36 (0.06,2.01) | 0.62 (0.02,16.81) | **EA3** |  |
| 0.07 (0.01,0.51) | 0.03 (0.00,3.10) | 0.16 (0.01,2.27) | 0.16 (0.00,19.06) | 0.26 (0.01,6.10) | 0.44 (0.10,1.92) | 0.72 (0.02,26.51 | **LA3** |

**RFA**: Radiofrequency Ablation with single treatment session**; RFA2**: Radiofrequency Ablation with 2 treatment session**; EA:** ethanol ablation with single treatment session**; EA3**: ethanol ablation with 3 treatment session; **LA**: Laser Ablation with single treatment session**; LA3**: Laser Ablation with 3 treatment session. **HIFU**: High-Intensity Focused Ultrasound.
